# Characteristics of hospital differences in missing of clinical laboratory test results in a multi-hospital observational database contributing to MID-NET® in Japan

**DOI:** 10.1186/s12911-021-01543-5

**Published:** 2021-06-06

**Authors:** Maki Komamine, Yoshiaki Fujimura, Yasuharu Nitta, Masatomo Omiya, Masaaki Doi, Tosiya Sato

**Affiliations:** 1 Department of Biostatistics, Kyoto University School of Public Health, Yoshida-konoecho, Sakyo-ku, Kyoto, 606-8501 Japan; 2 Office of Medical Informatics and Epidemiology, Pharmaceuticals and Medical Devices Agency, Tokyo, Japan; 3 Head Office, Tokushukai Information System Incorporated, Osaka, Japan; 4 Kishiwada Tokushukai Hospital, Osaka, Japan

**Keywords:** Drug safety, Clinical laboratory test, Database, Missing data, Observational study, Pharmacoepidemiology

## Abstract

**Background:**

In Japan, a multiple-hospital observational database system, the Medical Information Database Network (MID-NET®), was launched for post-marketing drug safety assessments. These assessments will be based on datasets with missing laboratory results. The characteristics of missing data considering hospital differences have not been evaluated. We assessed the missing proportion and the association between missingness and a factor through case studies using a database system, a part of MID-NET®.

**Methods:**

Seven scenarios using laboratory results before the prescription of the assessed drug as baseline covariates and data from 10 hospitals of Tokushukai Medical Group were used. The missing proportion and the association between missingness and patient background were investigated per hospital. The associations were assessed using the log of adjusted odds ratio (log-aOR). Additionally, an ad hoc survey was conducted to explore other factors affecting the missingness.

**Results:**

For some laboratory tests, missing proportions varied among hospitals, such as 7.4–44.4% of alkaline phosphatase (ALP) and 8.1–31.2% of triglyceride (TG) among statin users. The association between missingness and affecting factors also differed among hospitals for some factors; example, the log-aOR of hospitalization associated with missingness of TG was − 0.41 (95% CI, − 1.06 to 0.24) in hospital 3 and 1.84 (95% CI, 1.34 to 2.34) in hospital 4. In the ad hoc survey focusing on ALP, hospital-dependent differences in the ordering system settings were observed.

**Conclusions:**

Hospital differences in missing data appeared in some laboratory tests in our multi-hospital observational database, which could be attributed to the affecting factors, including the patient background.

**Supplementary Information:**

The online version contains supplementary material available at 10.1186/s12911-021-01543-5.

## Background

Observational databases, including health insurance claims and electronic medical records (EMRs), are crucial data sources for regulatory decision-making, providing clinical evidence on the usage and potential benefits or risks of a medical product [1–5]. Particularly, laboratory test results are useful sources of covariates or outcome measures in pharmacoepidemiological studies, including post-marketing drug safety assessments [6, 7].

The appropriate use of these data is difficult because some data obtained during routine medical care may be missing in datasets for analysis [8, 9]. Missing covariate data is a critical issue for observational studies requiring confounding adjustments. Various methods have been proposed to overcome improper handling of missing data that can result in a bias [8, 10]. The features of missing data (e.g., missing proportion and factors associated with missingness) and sources of missing data are crucial for choosing appropriate missing data methods [9, 10].

Missing proportions and factors associated with missingness can differ across data partners in databases covering multiple sites or hospitals. The variability in missing data among data partners is a critical issue for applying the missing data method. For three sites contributing to the US Food and Drug Administration Mini-Sentinel Distributed Database (MSDD), Raebel et al*.* [9] reported that the missing proportion of baseline laboratory results and factors associated with missingness varied by site. Differential missingness across sites was attributed to multiple factors, such as the type of data partner (e.g., only with laboratory results of outpatients) and patient background. The authors recommended applying a missing data method in a site-specific manner.

In Japan, the Medical Information Database Network (MID-NET®) was launched as a national project in April 2018 for post-marketing drug safety assessments [6, 7, 11]. This multi-hospital observational database system comprises 23 mid‐sized and large hospitals from 10 collaborative organizations [12]. Unlike those of the MSDD, all collaborative hospitals of the MID-NET® have EMRs as data sources of laboratory results. Hospital differences in missing laboratory results may still exist because of hospital-dependent potential factors (e.g., laboratory test measurement policies) and patient-dependent factors. Although laboratory results covered by the MID-NET® project are quality checked and standardized extensively [12], the features of missing data considering hospital differences have not been thoroughly evaluated.

We used data from 10 MID-NET®-collaborative hospitals and seven exposure-outcome scenarios using laboratory results as baseline covariates to investigate the characteristics of hospital differences in missing data as follows: (1) we investigated the frequency of laboratory result records and quantified the missing proportion; (2) we assessed the association between the missingness and a factor affecting missingness; and (3) we conducted an ad hoc survey to explore other factors affecting hospital differences in missing data. In some scenarios using laboratory results as outcome measures, we performed a supplementary investigation of the frequency of laboratory test records after the prescription date. For the detailed procedure and results (Additional file 1: Figure S1–S5), please refer to the Additional file 1.

## Methods

### Target hospitals and database

The MID-NET® is a distributed and closed network system in which each collaborative organization has a database system containing claims data, diagnosis procedure combination data, and EMRs [12]. The collaborative organizations consist of seven individual and three groups of hospitals’. Each group hospital database collectively stores data from their MID-NET®-contributing hospitals. The largest group hospital, Tokushukai Medical Group comprising 10 hospitals, was selected for investigating hospital differences with one database system.

The selected hospitals differ in size and serve as regional core hospitals with an emergency department. Hospital names are provided in Additional file 1: Table S1. We assigned hospital identification numbers 1–10 to ensure privacy in the results. EMRs in the database system for MID-NET®-collaborative organizations of Tokushukai Medical Group contain laboratory results, including those from the emergency department. The database does not capture hospital-specific data (e.g., laboratory test measurement policies and number of patients or beds).

### Definition of missing data

The observational database has two basic sources of missing laboratory results: a laboratory test was not conducted, and a laboratory test was conducted but not recorded [8, 9]. Because the two sources were difficult to distinguish, we defined missing data as follows: “data that would be meaningful for analysis but not available during a specific period.”

Missingness should be confirmed during a patient’s continuous consecutive observation. Therefore, we recreated the observation period for each patient by connecting hospital visits data. We then adopted two periods to confirm the missingness of laboratory results (the “target period”) as baseline covariates: (1) 90 days before the first prescription date (including the date) or (2) 180 days before the first prescription date (including the date). These periods were adopted by referring to previous cohort studies using a laboratory test as baseline covariate [13–15] and a previous study assessing missing data in the MSDD for 183 days [9].

### Frequency of laboratory result records and missing proportion

Frequencies of records in patients with laboratory result records of interest during a target period in each scenario were considered to assess the missing proportions. We counted the number of records per target period for each patient. Multiple records from the same day were out of the study objective and counted as one record. We then calculated the percentage of patients for each number of records in the overall cohort. The percentage of patients without a record, namely missing proportion, was also calculated for each hospital cohort.

### Association between missingness and a potential factor

We assessed hospital differences in the association between the missingness of laboratory result records before the prescription date and a potential factor affecting the missingness by fitting a logistic regression model in each hospital cohort of an individual scenario. Potential factors were selected based on previous cohort studies on each scenario [16–22] and a previous study assessing missing data in the MSDD [9]; they were sex, age, year of cohort entry, hospitalization, complications, concomitant medication, and class number of concomitant medications (see Additional file 1: Tables S2–S6 for individual factors). Complications or concomitant medications not observed in each hospital cohort were excluded from the covariates of hospital-specific logistic regression models. Each factor’s association was evaluated by the log of adjusted odds ratio (log-aOR) and 95% confidence interval (95% CI). In the model for scenario *l *$\left( {l = 1,{ } \ldots ,{ }L} \right)$, we used the following notation: $Y_{ijl}$, a missing data indicator (1 when missing or 0 otherwise); $X_{ijl}$, covariates; *i*, individuals of each hospital; *j*, number of laboratory tests; and $K_{l}$, number of covariates of each hospital. To estimate log-aORs, we fitted logistic models as 

$${\text{logit}}\left( {Pr(Y_{ijl} = 1|{\user2{X}}_{ijl} )} \right) = \upalpha + {\user2{X}}_{ijl}^{\prime } {\varvec{\beta}}_{l},$$

where $$\user2{ X}_{ijl} = \left( {X_{ij1l} , \ldots , X_{{ijK_{l} l}} } \right)^{\prime }$$.

### Scenarios

Seven cohort study scenarios using laboratory results as baseline covariates were created (Additional file 1: Figure S6). Scenarios 1–5 were original scenarios; scenarios 6 and 7 were incorporated to compare our results with those of Raebel et al*.* [9]. The backgrounds of the scenarios were as follows. Detail definition of each scenario’s cohorts is provided in Additional file 1: Figures S7–S13.

#### Scenario 1: Risk of diabetes associated with antipsychotic drug use

Glucose metabolism disorder is considered a risk of second-generation antipsychotics (SGAs) [16, 17]. We created a scenario with a cohort of new antipsychotic users to compare the diabetes risk of SGAs with that of first-generation antipsychotics (FGAs), considering blood glucose level and HbA1c as baseline covariates.

#### Scenario 2: Risk of hepatic injury associated with statin use

Hepatic injury is considered a risk common to all statins and mentioned in package inserts as a severe adverse effect. The attention level differs among statins (atorvastatin and rosuvastatin are contraindicated for patients with decreased liver function). Observational studies demonstrated that the hepatic injury risk of atorvastatin use, particularly that of high-dose use, is higher than that of other statins [23], and only a few studies indicated a similar risk in rosuvastatin and atorvastatin users [18]. We then created a scenario comparing the hepatic injury risk of atorvastatin with that of other statins, including rosuvastatin, considering low-density lipoprotein cholesterol (LDL-chol), triglyceride (TG), alanine aminotransferase (ALT), aspartate transaminase (AST), and alkaline phosphatase (ALP) as baseline covariates.

#### Scenario 3: Effect of uric acid synthesis inhibitor use on uric acid level

The uric acid-lowering effect of febuxostat was non-inferior to that of allopurinol in a Japanese phase III clinical trial [24]. Because patients with renal impairments were excluded from the trial’s target population, the effect on an overall population is unclear. We created a scenario comparing the uric acid-lowering effect of febuxostat with that of allopurinol, considering serum uric acid and serum creatinine as baseline covariates.

#### Scenario 4: Risk of hyponatremia associated with proton pump inhibitor use

Hyponatremia, a risk of lansoprazole use, is listed as a serious adverse effect in the lansoprazole package insert in Japan, but not in that of other proton pump inhibitors (PPIs). A case–control study indicated that other PPIs are associated with an increased hyponatremia risk [19]. We created a scenario comparing the hyponatremia risk of lansoprazole with that of other PPIs using serum sodium and serum creatinine as baseline covariates.

#### Scenario 5: Risk of acute pancreatitis associated with oral antidiabetic drug use

Acute pancreatitis is considered a risk of dipeptidyl peptidase-4 inhibitor (DPP-4I) use and listed in the DPP-4I package insert as a severe adverse effect in Japan. Some observational studies demonstrated that the acute pancreatitis risk associated with DPP-4Is may not be higher than that associated with other oral antidiabetic agents [20–22]. We created a scenario comparing the acute pancreatitis risk of DPP-4I with that of other oral antidiabetic agents, including biguanide, sulfonylurea, or α-glucosidase inhibitor, using blood glucose level, HbA1c, and serum amylase as baseline covariates.

#### Scenario 6: Risk of bleeding associated with the combination use of warfarin and an antimicrobial

Warfarin-related bleeding risk is managed by assessing anti-coagulability using international normalized ratio (INR). This laboratory result is affected by other drug use (interacting). Raebel et al*.* [9] created a scenario focusing on the concomitant use of warfarin with antimicrobial. Following their scenario setting, we created a scenario comparing the bleeding risk after antimicrobial use that can increase the INR with the bleeding risk after antimicrobial use that does not increase the INR, using the INR as a baseline covariate.

#### Scenario 7: Risk of diabetes associated with second-generation antipsychotic use

As mentioned in Scenario 1, glucose metabolism disorder is a risk of SGAs. Raebel et al*.* [9] created a scenario focusing on the risk of each SGA. Following their scenario setting, we created a scenario comparing the diabetes risk of olanzapine, quetiapine, or risperidone with that of aripiprazole, considering blood glucose level as a baseline covariate.

### Protocol approval and statistical analysis

Our study protocol was approved by the Kyoto University Graduate School and Faculty of Medicine Kyoto University Hospital Ethics Committee in November 2018 (R1793). Statistical analyses were performed using SAS version 9.4 (SAS Institute, Cary, NC, USA).

## Results

### Study cohorts

The overall cohorts were identified as follows: scenario 1: 3430 new antipsychotics users; scenario 2: 6195 new statin users; scenario 3: 3481 new users of uric acid synthesis inhibitors; scenario 4: 10,372 new PPI users; scenario 5: 2994 new users of oral antidiabetics; scenario 6: 965 new users of combinations of antimicrobials with warfarin; and scenario 7: 1007 new SGA users (Additional file 1: Figures S7–S13). Patient characteristics and their numbers in each hospital cohort are provided in Additional file 1: Tables S2–S6. The patient backgrounds differed among hospitals.

### Frequency of laboratory result records and missing proportion

**Fig. 1:**
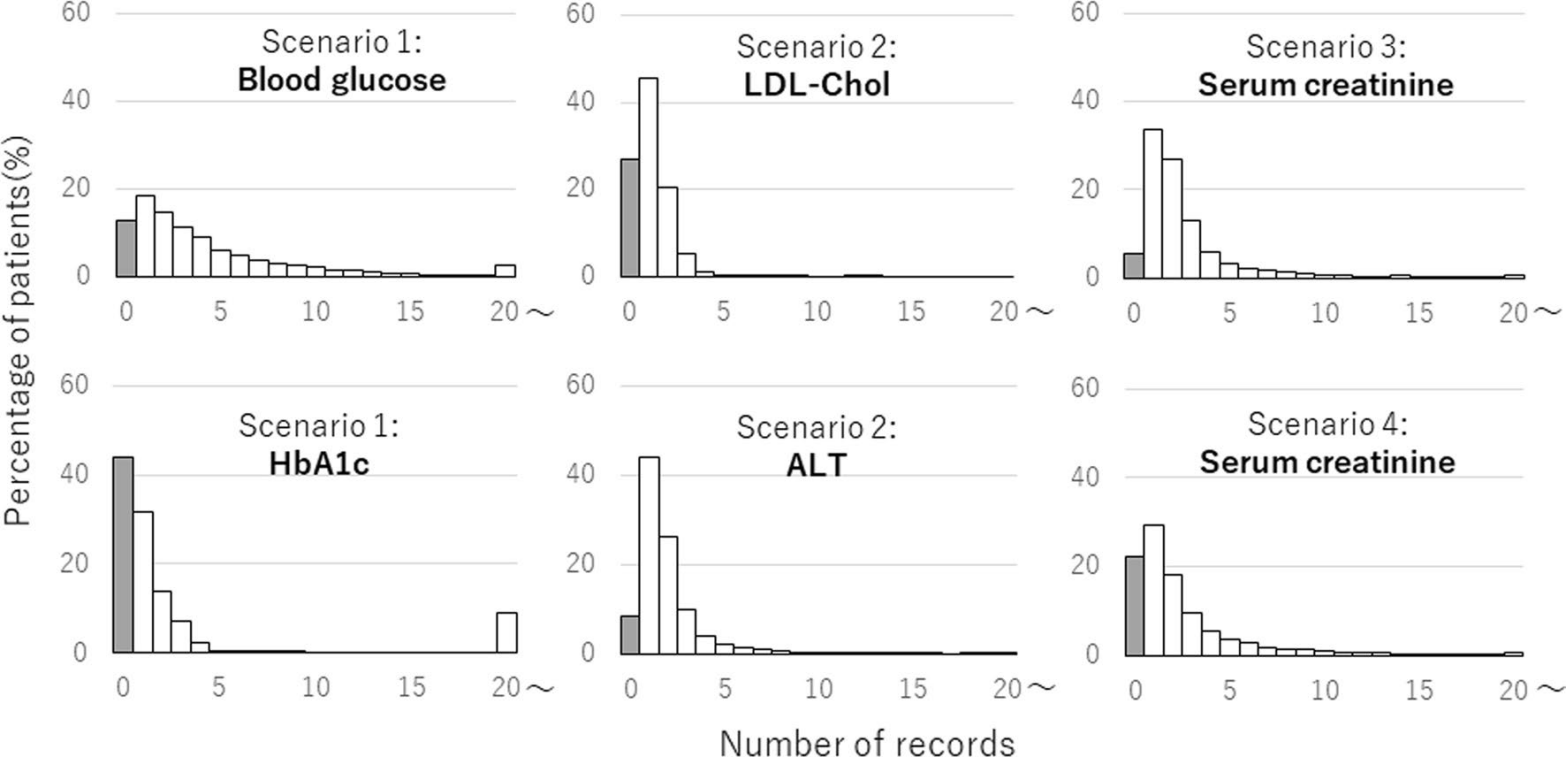
Variety of the frequency of laboratory results recorded within 90 days before prescription. This figure presents examples of the frequency of laboratory result records in the overall cohort, showing that the frequency and the missing proportions (indicated by shaded bars) differ among laboratory tests. *HbA1c* hemoglobin A1c, *LDL-chol* low-density lipoprotein cholesterol, *ALT* alanine transaminase

In the overall cohort, the frequency of laboratory result records within 90 days before prescription differed among laboratory tests (Fig. 1). In most laboratory tests, patients with one record were the most frequent, although some had multiple records (Table 1). In scenario 1, the percentage of patients with multiple records was higher for blood glucose than for HbA1c, 68.8% and 24.5%, respectively. The missing proportions (shaded bars, Fig. 1) were < 30%, except for HbA1c and serum amylase in scenarios 1 and 5. Extending the target period to 180 days changed these missing proportions within 7%.

**Table 1:** Frequency of laboratory result records and missing proportions in the overall cohort

Scenario no	Target laboratory test	Most frequent number of records	Percentage of patients with multiple records (%)	Missing proportion (%)	
		Period 1	Period 1	Period 1	Period 2
1	Blood glucose	1	68.8	12.8	9.9
	HbA1c	0	24.5	43.9	37.8
2	ALT	1	47.4	8.6	5.6
	AST	1	47.3	8.7	5.7
	ALP	1	31.6	29.2	24.8
	Bilirubin	1	38.5	20.0	16.2
	LDL-chol	1	27.6	27.0	23.1
	TG	1	30.9	20.0	16.5
3	Serum uric acid	1	49.2	13.2	10.6
	Serum creatinine	1	61.0	5.4	3.6
4	Serum sodium	1	46.0	25.9	20.6
	Serum creatinine	1	48.1	22.4	16.6
5	Blood glucose	1	59.2	4.0	2.7
	HbA1c	1	42.1	11.1	8.5
	Serum amylase	0	33.9	36.3	31.7
6	INR	2	70.2	9.7	5.7
7	Blood glucose	1	63.0	13.8	11.2

*HbA1c* hemoglobin A1c, *ALT* alanine transaminase, *AST* aspartate aminotransferase, *ALP* alkaline phosphatase, *LDL-chol* low-density lipoprotein cholesterol, *TG* triglyceride, *INR* international normalized ratio. Period 1 was 90 days before the first prescription date. Period 2 was 180 days before the first prescription date

**Fig. 2:**
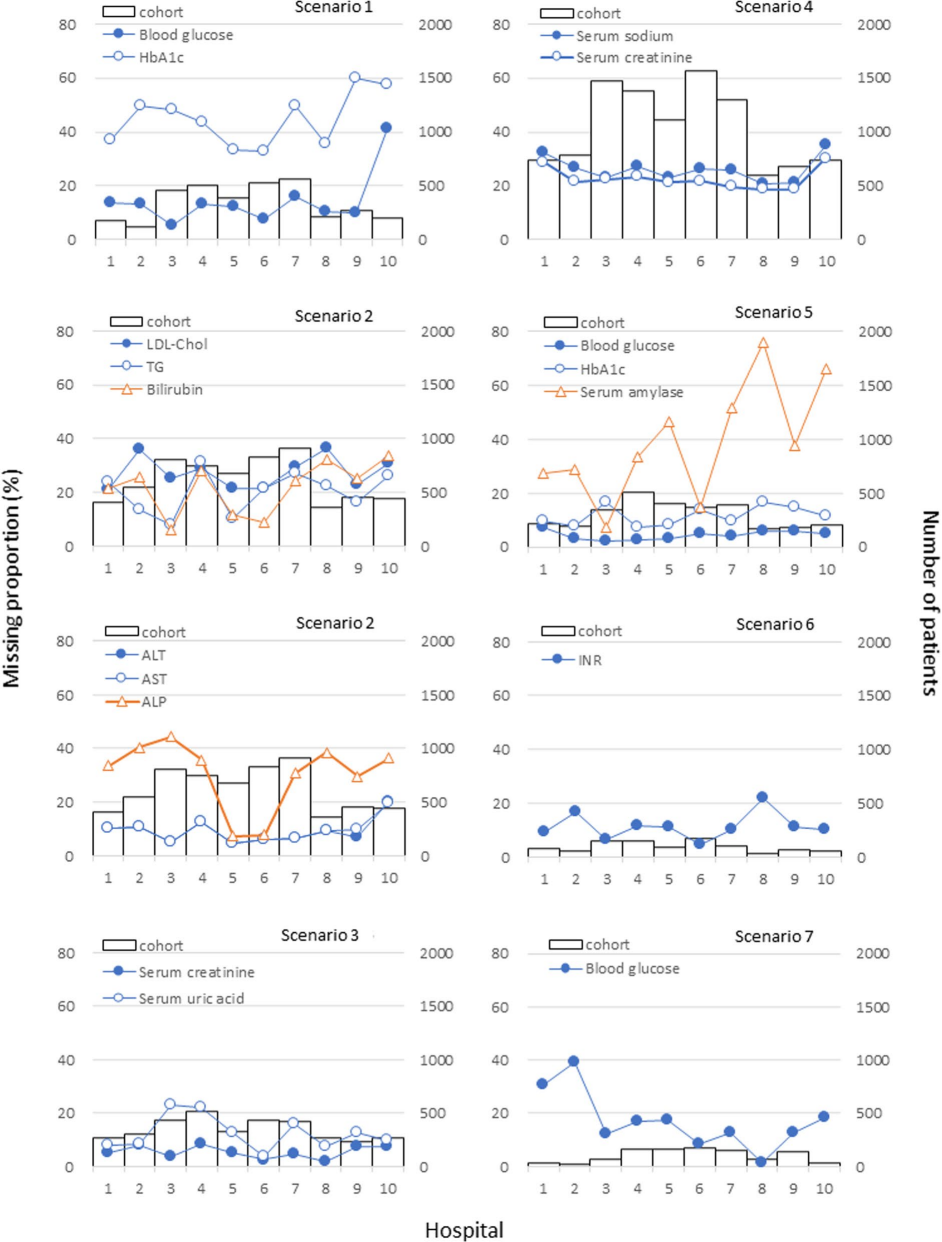
Missing proportion within 90 days before prescription in each hospital. This figure describes the differences in missing proportion (line graph) by hospital and number of patients (bar graph) among the hospital cohort. *HbA1c* hemoglobin A1c, *LDL-chol* low-density lipoprotein cholesterol, *TG* triglyceride, *ALT* alanine transaminase, *AST* aspartate aminotransferase, *ALP* alkaline phosphatase, *INR* international normalized ratio

In each hospital cohort, missing proportions within 90 days before prescription differed among hospitals for some laboratory tests; for example, 5.2–41.3% of blood glucose in scenario 1, 7.4–44.4% of ALP in scenario 2, 8.1–31.2% of TG in scenario 2, 4.7–21.9% of INR in scenario 6, 1.4–39.1% of blood glucose in scenario 7 (Fig. 2). In scenario 1, the blood glucose missing proportion was higher in hospital 10 than in the other hospitals. In scenario 2, the missing proportion variations of ALT/AST and ALP differed among the hospitals. Specifically, hospital 3 showed a large difference with 39.2% among these tests, whereas hospital 6 did not. Similar to the overall cohort results, extending the target period to 180 days did not substantially change the hospital differences; for example, 4.5–35.8% of blood glucose in scenario 1 and 5.4–39.3% of ALP (Additional file 1: Figure S14).

### Association between missingness and a potential factor

**Fig. 3:**
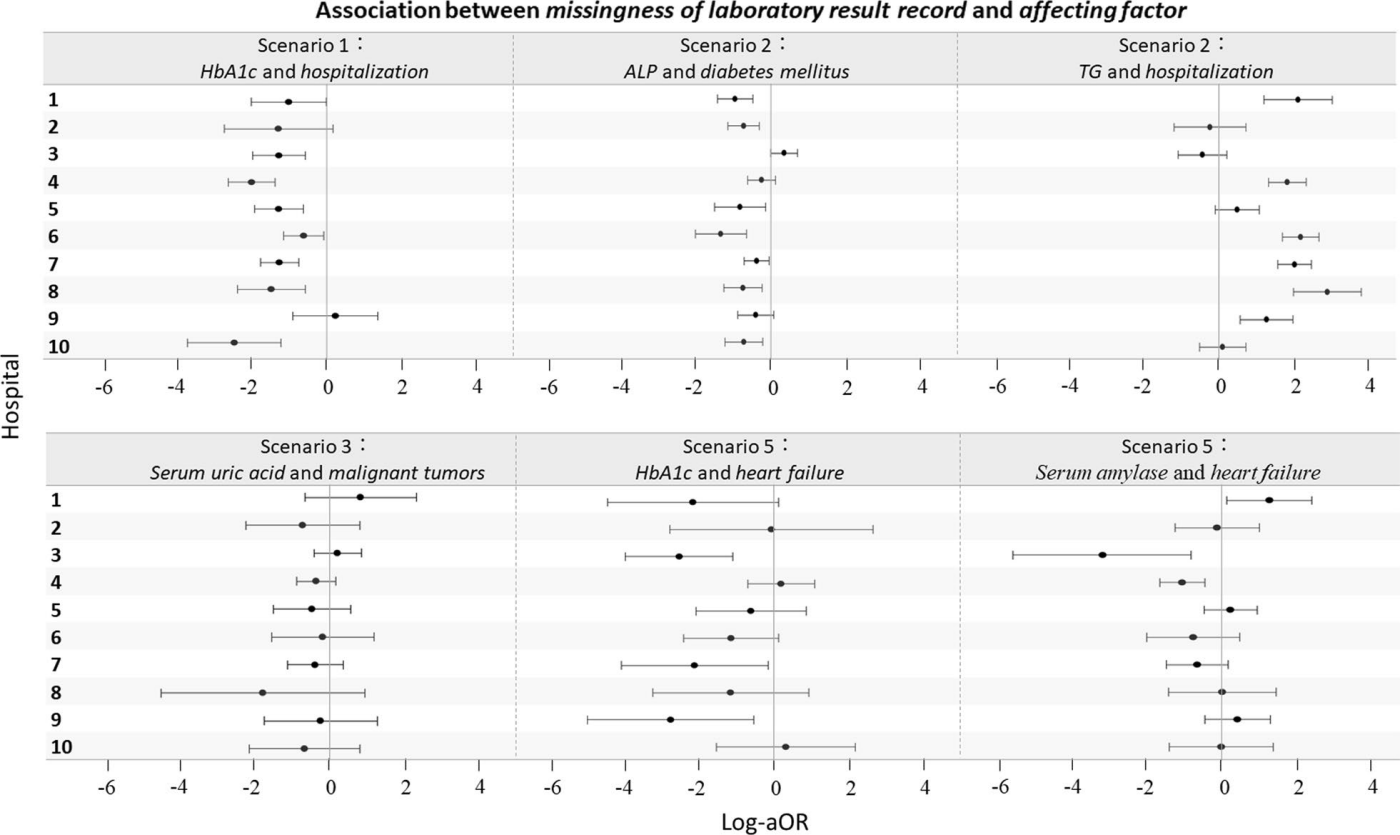
Varying associations between missingness and affecting factors among hospitals. This figure presents examples of the factors that have been suggested to affect hospital differences associated with missingness. *OR* odds ratio, *HbA1c* hemoglobin A1c, *LDL-chol* low-density lipoprotein cholesterol, *TG* triglyceride, *ALT* alanine transaminase, *ALP* alkaline phosphatase, *NSAIDs* non-steroidal anti-inflammatory drugs

Scenarios 6 and 7 were excluded from analysis because of the low patient numbers in the hospital cohorts. For some factors, the degree of association between missingness and the factor differed among hospitals (Fig. 3). For example, in scenario 2, the log-aOR of associating hospitalization with missingness of TG was < 0 in hospital 3 (log-aOR, − 0.41 [95% CI, − 1.06 to 0.24]) but > 0 in hospital 4 (log-aOR, 1.84 [95% CI, 1.34 to 2.34]).

Because hospital differences in the missing proportions within 180 days before prescription did not substantially vary from that within 90 days, this analysis was limited to the latter target period.

### Ad hoc survey

The missing proportion of ALT, AST, and ALP suggested an influence of hospital-dependent mechanical factors. The missing proportions may vary among these liver function tests because they measure different parameters. However, the degree of variation differed widely between hospitals 3 and 6.

Laboratory tests are ordered individually or in a group. Grouping can differ for each hospital because it can be customized. We assumed the effect of grouping on a chance of performing laboratory tests, namely missingness, and assessed the inclusion of ALT, AST, and ALP in groupings in hospitals 3 and 6 by confirming some groupings. We could not perform quantitative assessment and instead used the electronic laboratory ordering system because our database did not contain grouping data. We identified differences in some grouping settings; specifically, ALP was often grouped along with ALT or AST in hospital 6 but not in hospital 3.

## Discussion

We evaluated seven scenarios in a multi-hospital observational database system, a part of the MID-NET®, to investigate hospital differences in missing laboratory results for baseline covariates. In addition to these differences, we examined factors affecting the frequency of laboratory result records and missing data sources.

Variations in purpose for performing laboratory tests might have caused differences in the frequency of laboratory result records among laboratory tests or scenarios. In routine medical care, laboratory tests are performed to diagnose diseases and assess or monitor physiological functions [25]. For example, assessing and monitoring physiological functions could have contributed to regular laboratory testing and multiple records, such as serum creatinine in scenario 4. Variations in test intervals allowed by the health insurance in Japan (e.g., blood glucose, maximum of 60 per month for type 2 diabetes, and HbA1c, once per month) may have also affected the frequency. The period for confirming the missingness should be created considering these factors and the study objective.

Several factors contributed to missing laboratory results in our database. Few studies have systematically referred to missing data sources, except the MSDD-based study by Raebel et al*.* [9] In the MSDD, the missing data sources included type of data partner, patient location where tests were conducted (e.g., emergency department), collectability from outside of contracted laboratories, and patient backgrounds. Our database had some common and different sources compared to this previous study. Patient backgrounds were considered to affect the missing data in our database, similar to observations in the previous study. However, the contribution of the other three factors to the missing data may be limited, although this was not quantitatively assessed. All 10 hospitals in our study are the same type of data partner and had EMR-based laboratory results, including those of the emergency department. Laboratory tests assessed were mainly performed in the hospital and not outsourced. A new potential source was the grouping of laboratory tests. Other remaining potential factors included the policy for performing laboratory tests, which was considered at the planning stage but not assessed because of a lack of data.

Our database had hospital differences in the missing proportion and association between the missingness and a factor affecting missingness. As described above, there were few missing data sources in our database. Patient backgrounds were a substantial source, and the grouping of laboratory tests to order remains a potential source. In some patient backgrounds, the association with the missingness differed among hospitals. Additionally, the range of hospital differences in missing blood glucose in scenario 1 was reduced from 36.1 to 11.7% by limiting the study subjects to patients over 21 years of age in the additional analysis. In the ad hoc survey focusing on ALP with substantial hospital differences in missing proportion, hospital-dependent differences in the setting of some groupings of laboratory tests were observed. In our database, hospital-dependent potential missing data sources exist, but the corresponding data are not available for analysis. Therefore, missing data methods should consider hospital- effects (such as using a hospital-specific approach).

Variations in the type of missing data sources among databases accounted for the difference in the missing proportion. In scenarios 6 and 7, differences among hospitals were lower than those among sites in a previous study [9] (INR from scenario 6: 2.8–21.9% vs. approximately 8.0–80.0%; blood glucose from scenario 7: 1.4–30.6% vs. 41.1–72.3%). Although study population differences caused these variations, the differences in missing data sources among databases may have also contributed.

This study had several strengths. First, we investigated the characteristics of hospital differences in missing laboratory results using a part of the MID-NET®. As these characteristics also exist in the entire MID-NET®, our findings will provide guidance for using MID-NET®, which is a national project. Second, we observed hospital differences in the missing data and discussed the missing data source affecting these differences: patient background and grouping of laboratory tests to order. Finally, we observed various missing proportions by including multiple laboratory tests. The variations contributed to characterizing the hospital differences in missing data; although, the laboratory tests used were limited.

Nonetheless, there were some limitations. First, laboratory tests not covered by our study may have other missing data characteristics. Second our results may not be generalizable to the entire MID-NET®. There were differences among the 10 Tokushukai Medical Group hospitals that exist in the entire MID-NET®. However, a non-difference observed among the 10 hospitals does not assure it is a non-difference in the entire MID-NET®. Other hospitals may have different factors affecting the missing proportion or their hospital differences. For example, the 10 Tokushukai Medical Group hospitals are mainly general hospitals, whereas the other hospitals are mostly specialized hospitals. As the latter provides medical care to patients referred from other hospitals and clinics, referral rates may be a factor.

## Conclusions

We conclude that hospital differences in the missing data appeared in some laboratory tests in a multiple-hospital observational database system contributing to the MID-NET® because of factors such as patient background, although all hospitals are the same type of data partner. Importantly, these differences were found in the entire MID-NET®. As the data of hospital-dependent factors affecting missingness are not available in MID-NET®, missing data methods should be applied while considering the effect of each hospital (e.g., use a hospital-specific approach). Further studies should investigate the influence of these hospital differences on outcome parameter estimations.

## Supplementary Information

**Additional file 1**. Supplemental investigation.

## Data Availability

Based on the terms of use for MID-NET® to which we adhered when conducting this study, the dataset used for analysis cannot be made openly available; the accessibility of the dataset used for this analysis is restricted to specific researchers in a predetermined secure environment. No outside researchers are allowed to access the dataset.. This study used the database system for MID-NET®-collaborative organizations of the Tokushukai Medical Group, a part of MID-NET®, and not the entire MID-NET®. However, we followed the terms of use for MID-NET®, as the datasets were included in the entire MID-NET®.

## Abbreviations

ALP: Alkaline phosphatase
ALT: Alanine aminotransferase
AST: Aspartate transaminase
CI: Confidence interval
DPP-4I: Dipeptidyl peptidase-4 inhibitor
EMR: Electronic medical record
FGA: First-generation antipsychotic
INR: International normalized ratio
LDL-chol: Low-density lipoprotein cholesterol
Log-aOR: Log of adjusted odds ratio
MID-NET: Medical Information Database Network
MSDD: Mini-Sentinel Distributed Database
NGSP: National Glycohemoglobin Standardization Program
PPI: Proton pump inhibitor
SGA: Second-generation antipsychotic
TG: Triglyceride
