# Supplementary material for: Characteristics of hospital differences in missing of clinical laboratory test results in a multi-hospital observational database contributing to MID-NET® in Japan
Source: BMC Med Inform Decis Mak. 2021 Jun 6;21:181. doi: 10.1186/s12911-021-01543-5 (PMC8180009; doi:10.1186/s12911-021-01543-5)
Supplement: Supplementary file 1 — Additional file 1. Supplemental investigation. [file 12911_2021_1543_MOESM1_ESM.docx]

# **Supplemental investigation:**

**Methods**

Depending on the study’s interests, the target population may comprise patients without diabetes based on the baseline laboratory results. Thus, we also created a sub-cohort that only included patients confirmed to be diabetes-free using baseline blood glucose or HbA1c (National Glycohemoglobin Standardization Program; NGSP) as follows: patients with a blood glucose level of 200 mg/dL or HbA1c(NGSP) level of 6.5%, or without a record of blood glucose and HbA1c, were excluded.

*Laboratory tests as the outcome measure:*

　Scenario 1: Blood glucose, HbA1c

　Scenario 2: ALT, AST, ALP

　Scenario 3: Serum uric acid

　Scenario 4: Serum sodium

*Target period in the definition of missing data:*

We adopted three periods for the outcomes: 1) period from the prescription date to the observation period end, 2) period from 365 days after the first prescription date, or 3) 84 days after the first prescription date. The first and second periods were adopted for cases in which all outcomes were included and for cases in which the study interest was the only outcome after a certain period from the prescription date, respectively. The 365 days in the fourth period was created by referring to the mean follow-up period in a previous study of our scenario.^1^ The last period was adopted for scenario 3, considering the follow-up period used in clinical trials (8 weeks) and different treatment intervals for each patient.^2^

*Frequency of laboratory result records and missing proportion:*

We counted the number of records per target period and calculated the percentage in the overall cohort. In the analysis using the first target period, we calculated the quartiles, along with the maximum and minimum values of the period, because of the variations in the patient-dependent target period.

**Results**

**
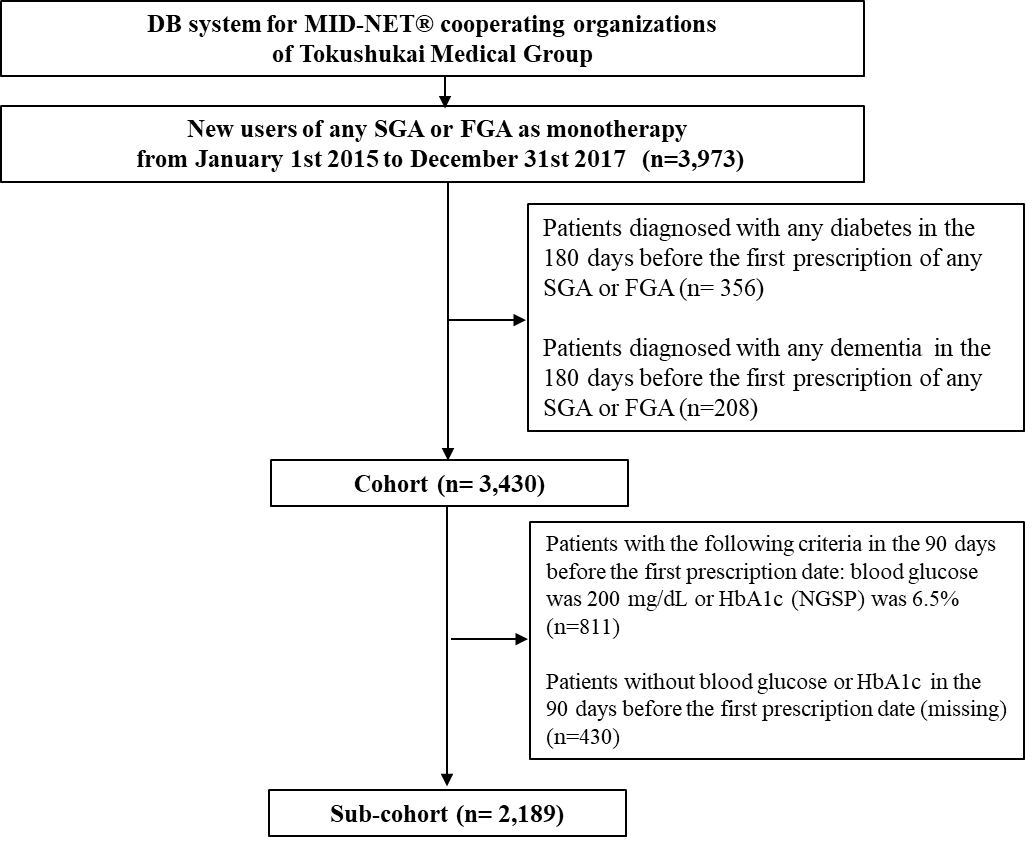
**

# **Supplementary Figure S1. Number of patients in the study sub-cohort of scenario 1.**


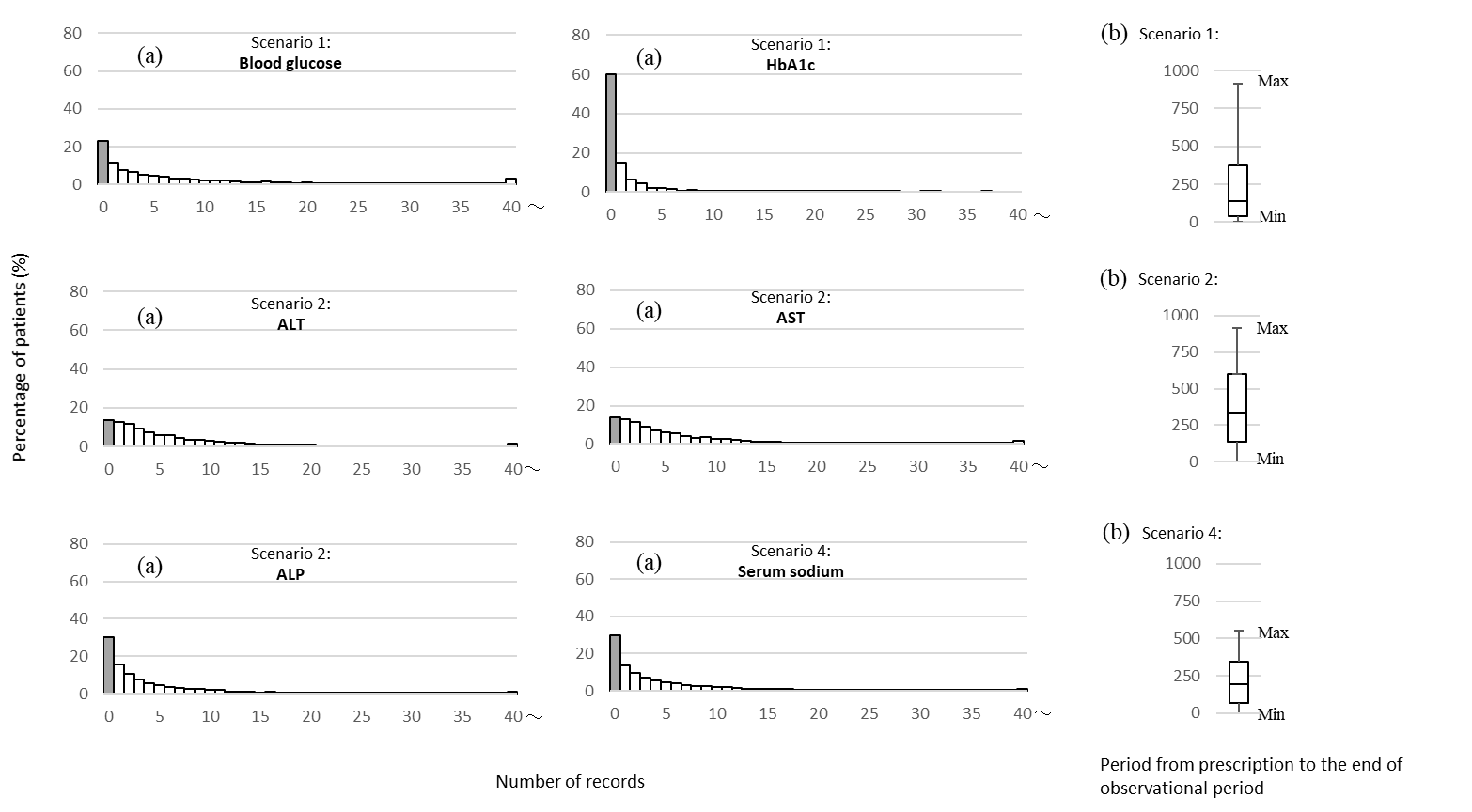


# **Supplementary Figure S2. Frequency of laboratory results recorded from prescription to the end of observation period in the overall cohort in scenarios 1, 2, and 4.**

The frequency of laboratory result records after prescription differed from that before prescription. For example, in ALT of scenario 2, the percentage of patients with only one remarkable record decreased from 44.0% to 12.8%, and the missing proportion increased from 8.6% to 13.8%.

**
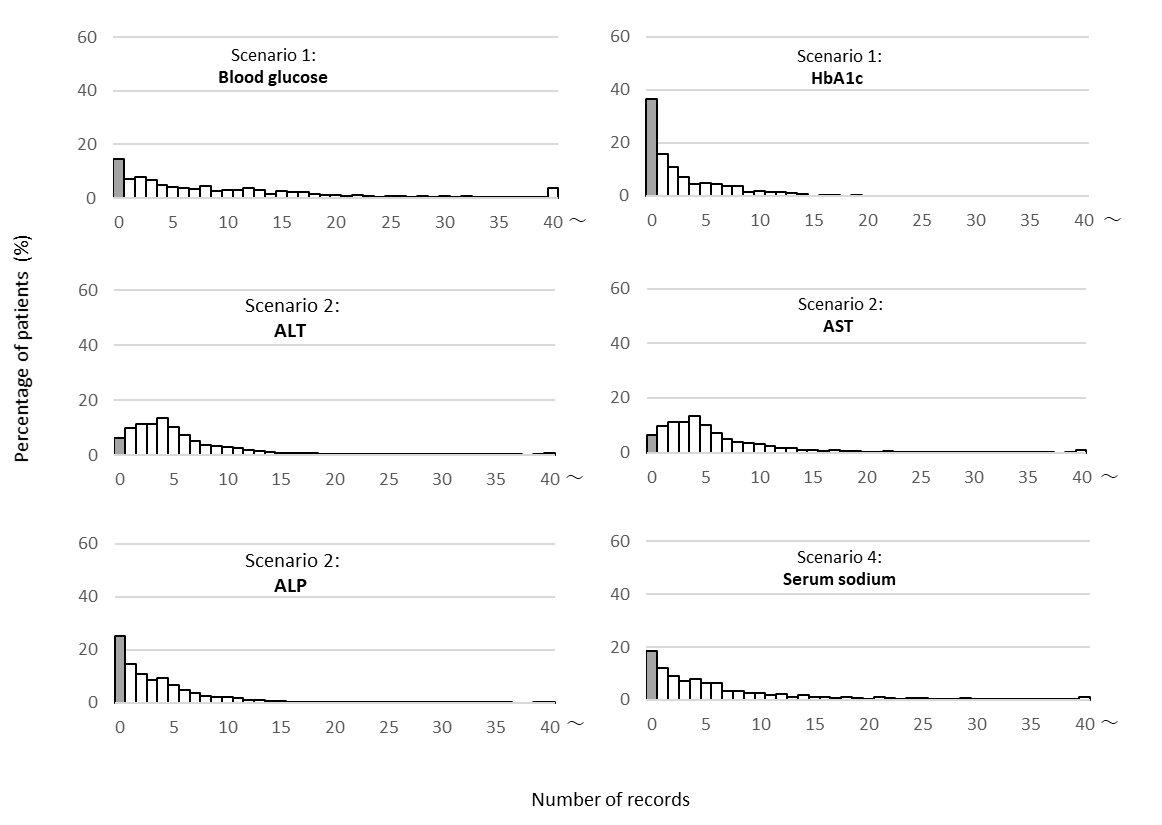
**

# **Supplementary Figure S3. Frequency of records of laboratory tests within 365 days after prescription in the overall cohort in scenarios 1, 2, and 4.**

Limiting the target period to 365 days improved the missing proportion despite a decrease in patient numbers: scenario 1: 881 patients; scenario 2: 2901 patients; and scenario 4: 2333 patients. For example, the ALT missing proportion was 6.1%, which was 7.7% lower than that without restriction for the target period.


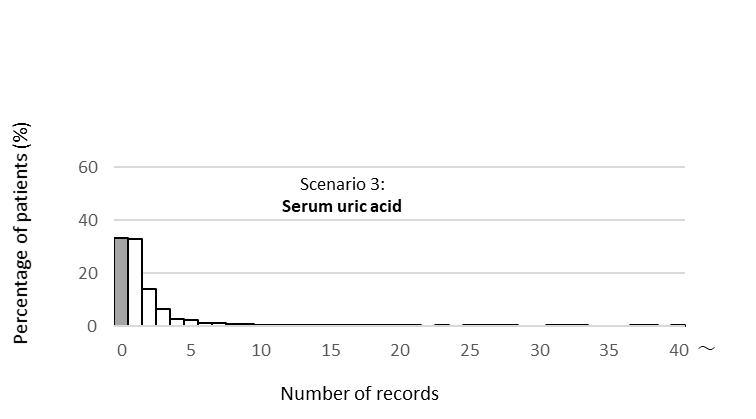


# **Supplementary Figure S4. Frequency of laboratory results recorded within 84 days after prescription in the overall cohort.**

In scenario 3, the missing proportion was 33.2% within 84 days after prescription.


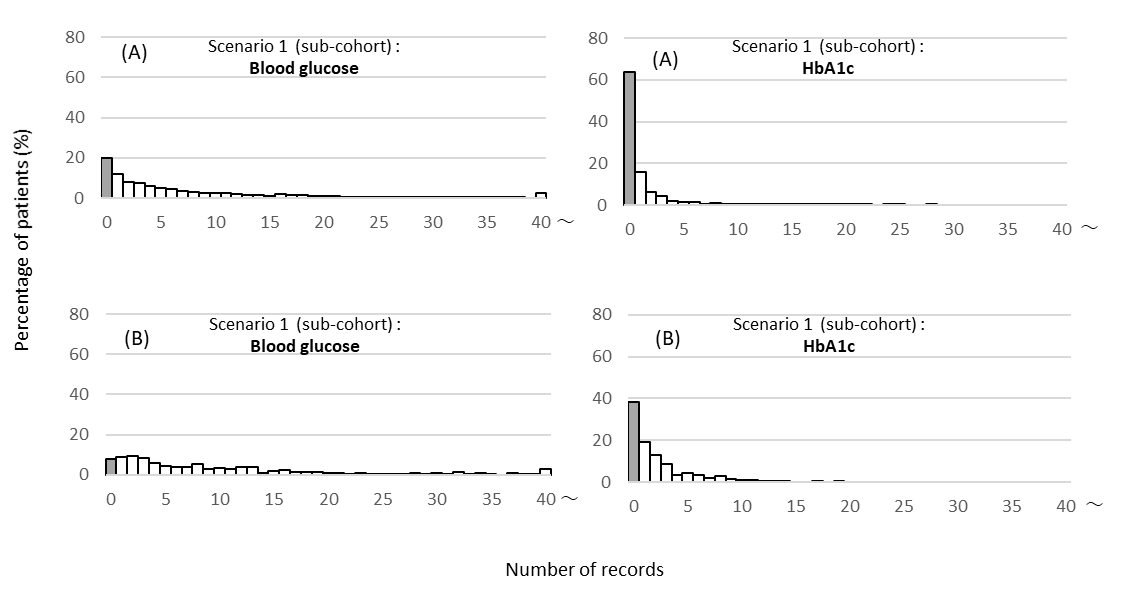


# **Supplementary Figure S5. Summary of results among the sub-cohort of scenario 1:**

1. Number of records of laboratory tests in the period from the prescription date to the end of the observational period. (B) Number of records of laboratory tests within 365 days after prescription.

In scenario 1, the blood glucose missing proportion among the sub-cohort was 19.9%, which was 3.1% lower than that among the cohort.

**Reference**

1. Clarke AT, Johnson PC, Hall GC, Ford I, Mills PR. High dose atorvastatin associated with increased risk of significant hepatotoxicity in comparison to simvastatin in UK GPRD cohort. PLoS One. 2016;11:e0151587.
2. Pharmaceuticals and Medical Devices Agency: Review report of febuxostat. <http://www.pmda.go.jp/files/000223354.pdf>; 2010 Accessed 31 Jul 2020.

# **Supplementary Tables and Figures referenced in the manuscript:**

# **Supplementary Table S1. List of 10 hospitals in the database system for MID-NET® collaborative organizations of Tokushukai Medical Group**

| Uji Tokushukai Hospital |
| --- |
| Kishiwada Tokushukai Hospital |
| Sapporo Tokushukai Hospital |
| Shonan Fujisawa Tokushukai Hospital |
| Tokyo-Nishi Tokushukai Hospital |
| Nagoya Tokushukai General Hospital |
| Nozaki Tokushukai Hospital |
| Fukuoka Tokushukai Hospital |
| Matsubara Tokushukai Hospital |
| Yao Tokushukai General Hospital |

# **Supplementary Table S2. Patient characteristics in the scenario 1 cohort:**

|  | All hospitals combined | Individual hospital | | | | | | | | | |
| --- | --- | --- | --- | --- | --- | --- | --- | --- | --- | --- | --- |
|  |  | 1 | 2 | 3 | 4 | 5 | 6 | 7 | 8 | 9 | 10 |
| **New users (n)** | 3,430 | 174 | 121 | 463 | 505 | 388 | 531 | 563 | 208 | 276 | 201 |
| **Sex, male**^‡^ **(%)** | 58.7 | 54.0 | 58.7 | 64.8 | 59.2 | 57.7 | 58.2 | 60.0 | 43.3 | 56.5 | 65.2 |
| **Age**^‡^, **mean (SD)** | 71.2 (17.7) | 70.7 (15.5) | 75.9 (11.1) | 71.3 (13.9) | 70.2 (19.5) | 70.1 (17.7) | 71.4 (15.4) | 74.2 (12.6) | 75.1 (17.3) | 76.1 (13.6) | 52.8 (32) |
| **Year of cohort entry**^‡^ |  |  |  |  |  |  |  |  |  |  |  |
| 2015 | 19.0 | 16.7 | 19.0 | 19.7 | 19.8 | 17.5 | 18.5 | 19.9 | 25.5 | 17.8 | 14.9 |
| 2016 | 41.9 | 35.6 | 32.2 | 43.2 | 46.7 | 40.7 | 42.6 | 43.0 | 35.1 | 44.6 | 38.8 |
| 2017 | 39.1 | 47.7 | 48.8 | 37.1 | 33.5 | 41.8 | 39.0 | 37.1 | 39.4 | 37.7 | 46.3 |
| **Hospitalization**^‡^ **(%)** | 74.4 | 66.7 | 75.2 | 79.5 | 72.5 | 73.7 | 81.4 | 66.3 | 77.4 | 92.8 | 51.7 |
| **Number of ambulatory medical visits^†§^**, **mean (SD)** | 10.5 (10.9) | 9.3 (5.8) | 10 (11.2) | 10.9 (12.1) | 10.8 (9.4) | 9.6 (9.1) | 12.5 (14.6) | 9.3 (8.6) | 10.5 (13.4) | 9.9 (10.4) | 10 (9.6) |
| **Number of hospitalizations^†§^**, **mean (SD)** | 1.2 (1) | 1.1 (1.4) | 1.2 (0.9) | 1.3 (0.9) | 1.2 (0.9) | 1.1 (0.9) | 1.3 (0.9) | 1.1 (0.9) | 1.2 (0.9) | 1.7 (1.2) | 0.8 (1) |
| **Class number of any prescriptions^†§^**, **mean (SD)** | 18.9 (10.2) | 20.4 (11) | 18.6 (9.8) | 21.9 (12.4) | 19.6 (10.6) | 17.4 (9) | 21.2 (9) | 15.4 (7.9) | 20.4 (10.2) | 19.9 (9.3) | 12.1 (8.6) |
| **Class number of concomitant medications^§^**, **mean (SD)** | 0.7 (0.8) | 0.7 (0.8) | 0.7 (0.8) | 0.9 (0.9) | 0.8 (1) | 0.7 (0.8) | 0.8 (0.8) | 0.6 (0.8) | 0.6 (0.8) | 0.7 (0.8) | 0.4 (0.7) |
| **Exposure (%)** |  |  |  |  |  |  |  |  |  |  |  |
| FGAs | 68.3 | 72.4 | 74.4 | 90.5 | 65.5 | 54 | 68.4 | 73.5 | 66.8 | 55.4 | 48.8 |
| SGAs | 31.7 | 27.6 | 25.6 | 9.5 | 34.5 | 45.9 | 31.6 | 26.5 | 33.2 | 44.6 | 51.2 |
| **Complications^§^ (%)** |  |  |  |  |  |  |  |  |  |  |  |
| Hepatitis | 4.0 | NA | NA | 5.4 | 5.1 | 4.1 | 3.2 | 2.1 | 4.8 | 5.4 | NA |
| Liver cirrhosis | 1.9 | NA | NA | NA | 2.6 | 3.6 | 1.9 | NA | NA | NA | NA |
| Chronic pancreatitis | 0.5 | NA | 0.0^†^ | NA | NA | 0.0^†^ | NA | NA | NA | NA | NA |
| Hypertension | 11.8 | 11.5 | 13.2 | 12.7 | 11.5 | 8.0 | 14.5 | 12.1 | 12.5 | 11.2 | 9.0 |
| Hyperlipidemia | 6.7 | NA | NA | 4.8 | 4.0 | 8.0 | 5.8 | 12.1 | 6.3 | 7.2 | 6.0 |
| Hyperthyroidism | 3.0 | NA | NA | NA | 2.4 | 2.6 | 6.4 | NA | 4.8 | 5.1 | NA |
| Cushing’s syndrome | 1.0 | 0.0^†^ | 0.0^†^ | NA | 0.0^†^ | 7.5 | NA | NA | 0.0^†^ | 0.0^†^ | 0.0^†^ |
| Primary aldosteronism | NA | 0.0^†^ | 0.0^†^ | 0.0^†^ | 0.0^†^ | 0.0^†^ | 0.0^†^ | NA | 0.0^†^ | 0.0^†^ | 0.0^†^ |
| Pancreatic cancer | 2.7 | NA | NA | 5.0 | 3.4 | NA | 2.3 | 2.3 | NA | NA | NA |
| Liver cancer | 5.0 | 7.5 | NA | 4.3 | 6.3 | 5.9 | 3.4 | 5.7 | 10.6 | NA | NA |
| Pheochromocytoma | NA | 0.0^†^ | 0.0^†^ | 0.0^†^ | 0.0^†^ | NA | 0.0^†^ | 0.0^†^ | 0.0^†^ | 0.0^†^ | 0.0^†^ |
| Hemochromatosis | NA | 0.0^†^ | NA | 0.0^†^ | 0.0^†^ | 0.0^†^ | 0.0^†^ | 0.0^†^ | 0.0^†^ | 0.0^†^ | 0.0^†^ |
| Schizophrenia | 12.2 | 6.3 | 24.0 | 5.8 | 15.4 | 8.5 | 10.9 | 19.7 | 11.1 | 5.4 | 17.4 |
| Mood disorder | 4.4 | NA | NA | 4.8 | 3.6 | 7.0 | 4.3 | 4.4 | 8.2 | NA | NA |
| Neurotic disorder | 5.1 | NA | 8.3 | 4.5 | 4.2 | 2.8 | 4.0 | 9.8 | 7.7 | NA | NA |
| Cancer other than liver and pancreatic | 42.2 | 25.9 | 42.1 | 46.4 | 48.9 | 34.3 | 45.2 | 46.2 | 43.3 | 38.8 | 29.9 |
| **Concomitant medication^§^ (%)** |  |  |  |  |  |  |  |  |  |  |  |
| Beta-blockers | 19.9 | 22.4 | 20.7 | 26.6 | 19.8 | 24.0 | 19.8 | 13.0 | 15.4 | 25.0 | 11.4 |
| Thiazide diuretics | 3.8 | NA | NA | 3.0 | 7.1 | 4.9 | 2.8 | 2.1 | 5.3 | 4.3 | NA |
| Antidepressants | 8.5 | 13.2 | 8.3 | 8.9 | 5.9 | 15.5 | 10.0 | 6.6 | 7.7 | NA | 7.0 |
| Corticosteroids | 26.6 | 18.4 | 21.5 | 32.6 | 31.1 | 18.3 | 29.6 | 28.8 | 21.2 | 29.3 | 15.9 |
| Interferon prepared | NA | NA | 0.0^†^ | 0.0^†^ | 0.0^†^ | 0.0^†^ | 0.0^†^ | 0.0^†^ | 0.0^†^ | 0.0^†^ | 0.0 |
| High-calorie transfusion agents | 5.7 | NA | 9.1 | 7.1 | 9.1 | 4.6 | 6.0 | 3.0 | 7.7 | 4.7 | NA |
| Immunosuppressant | 0.8 | 0.0^†^ | 0.0^†^ | NA | NA | NA | NA | NA | NA | NA | NA |
| **First visit**^‡^ **(%)** | 1.3 | NA | NA | NA | NA | NA | NA | 1.8 | NA | NA | NA |
| **Emergency care**^‡^ **(%)** | 27.8 | 24.7 | 34.7 | 34.1 | 43.4 | 18.8 | 25.2 | 27.0 | 23.1 | 17.8 | 16.9 |

Some categories in which the patient numbers did not exceed 10 were not shown due to privacy.

NA is the case when the number of patients was less than 10.

Abbreviations: FGA, first-generation antipsychotic; SGA, second-generation antipsychotic.

† Not included in logistic regression model for assessing the association with missingness.

‡ At the date of the first prescription of any antidiabetic drug.

§ 180 days prior to the date of the first prescription of any antidiabetic drug.

# **Supplementary Table S3. Patient characteristics in the scenario 2 cohort:**

|  | All hospitals combined | Individual hospital | | | | | | | | | |  |
| --- | --- | --- | --- | --- | --- | --- | --- | --- | --- | --- | --- | --- |
|  |  | 1 | 2 | 3 | 4 | 5 | 6 | 7 | 8 | 9 | 10 | |
| **New users (n)** | 6,195 | 409 | 545 | 807 | 753 | 679 | 831 | 911 | 360 | 456 | 444 | |
| **Sex, male**^‡^ **(%)** | 53.6 | 49.9 | 50.8 | 57.4 | 52.9 | 54.2 | 54.9 | 54.7 | 41.9 | 59.2 | 53.6 | |
| **Age**^‡^, **mean (SD)** | 69.5 (12.2) | 68.1 (12.7) | 68.9 (11.9) | 70.4 (11.0) | 69.2 (12.7) | 68.8 (12.5) | 69.3 (12) | 71.2 (12) | 67.9 (12.6) | 70.2 (12.3) | 69 (12.7) | |
| **Year of cohort entry**^‡^**(%)** |  |  |  |  |  |  |  |  |  |  |  | |
| 2015 | 21.2 | 23.5 | 23.9 | 19.0 | 26.4 | 21.1 | 21.2 | 20.9 | 19.2 | 18.0 | 16.7 | |
| 2016 | 39.2 | 35.9 | 40.2 | 38.7 | 38.5 | 40.9 | 35.0 | 41.4 | 37.5 | 45.2 | 38.5 | |
| 2017 | 39.7 | 40.6 | 36.0 | 42.4 | 35.1 | 38.0 | 43.8 | 37.8 | 43.3 | 36.8 | 44.8 | |
| **Hospitalization**^‡^ **(%)** | 38.6 | 14.9 | 15.8 | 43.9 | 38.6 | 50.1 | 47.9 | 53.1 | 25.6 | 28.1 | 35.6 | |
| **Number of ambulatory medical visits^†§^**, **mean (SD)** | 6.4 (6.2) | 6.5 (4.8) | 6.2 (6.1) | 5.9 (4.3) | 6.7 (5.0) | 6.1 (4.8) | 7 (7.8) | 6.2 (6.6) | 6.8 (7.8) | 6.5 (6.9) | 6.6 (7.9) | |
| **Number of hospitalizations^†§^**, **mean (SD)** | 0.6 (0.7) | 0.3 (0.7) | 0.3 (0.7) | 0.7 (0.7) | 0.5 (0.6) | 0.6 (0.6) | 0.7 (0.6) | 0.7 (0.7) | 0.4 (0.9) | 0.5 (0.8) | 0.6 (0.9) | |
| **Class number of any prescriptions^†§^**, **mean (SD)** | 10.1 (6.6) | 9.4 (6.8) | 9.1 (6.2) | 11.4 (7.3) | 10.7 (6.9) | 10.5 (6.4) | 10.6 (6.7) | 9.6 (5.8) | 9.4 (6.3) | 10.4 (7.4) | 9.0 (6.0) | |
| **Class number of concomitant medications^§^**, **mean (SD)** | 0.5 (0.7) | 0.6 (0.7) | 0.5 (0.6) | 0.5 (0.6) | 0.5 (0.7) | 0.4 (0.6) | 0.5 (0.7) | 0.4 (0.6) | 0.5 (0.7) | 0.6 (0.8) | 0.5 (0.7) | |
| **Exposure (%)** |  |  |  |  |  |  |  |  |  |  |  | |
| Atorvastatin | 25.5 | 19.6 | 0.0 | 23.8 | 27.1 | 23.7 | 41.0 | 27.7 | 36.4 | 22.6 | 26.4 | |
| Rosuvastatin | 47.5 | 39.4 | 74.1 | 41.8 | 56.7 | 52.6 | 38.6 | 44.9 | 41.9 | 39.0 | 44.4 | |
| Simvastatin | 0.8 | NA | 0.0 | 1.4 | 2.1 | NA | NA | 1.1 | NA | 0.0 | 0.0 | |
| Fluvastatin | 0.6 | 0.0 | 0.0 | NA | NA | 1.5 | NA | NA | NA | NA | NA | |
| Pitavastatin | 13.5 | 17.6 | 22.6 | 21.4 | 3.2 | 8.5 | 3.1 | 14.4 | 7.2 | 28.1 | 17.1 | |
| Pravastatin | 12.1 | 23.2 | 3.3 | 11.2 | 10.4 | 13.1 | 16.1 | 11.1 | 13.6 | 10.1 | 11.0 | |
| **Complications^§^ (%)** |  |  |  |  |  |  |  |  |  |  |  | |
| Chronic kidney disease | 2.7 | NA | NA | 3.8 | 1.5 | 6.8 | 2.3 | 2.6 | 3.6 | NA | NA | |
| Heart failure | 25.4 | 31.8 | 19.4 | 39.3 | 21.6 | 24.4 | 22.5 | 17.2 | 27.2 | 33.1 | 21.8 | |
| Acute myocardial infarction | 11.2 | 18.1 | 4.0 | 11.6 | 8.6 | 13.0 | 8.4 | 13.4 | 13.1 | 14.3 | 11.0 | |
| Hypertension | 20.3 | 17.1 | 15.4 | 20.0 | 21.6 | 18.1 | 23.1 | 24.0 | 13.9 | 24.1 | 18.9 | |
| Cerebrovascular diseases | 10.8 | 11.0 | 11.2 | 11.2 | 13.1 | 12.8 | 12.8 | 11.3 | 3.9 | 7.2 | 7.7 | |
| Diabetes mellitus | 45.0 | 52.8 | 33.9 | 49.2 | 37.8 | 46.1 | 43.4 | 51.8 | 53.6 | 43.0 | 37.8 | |
| Peripheral vascular disease | 0.6 | NA | NA | 1.2 | NA | NA | NA | NA | NA | 0.0^†^ | NA | |
| **Concomitant medication^§^ (%)** |  |  |  |  |  |  |  |  |  |  |  | |
| Antiepileptic drugs | 0.6 | NA | NA | NA | NA | NA | NA | NA | 0.0^†^ | NA | 0.0^†^ | |
| Fibrates | 3.5 | 4.4 | 6.2 | 2.9 | 2.4 | NA | 2.4 | 2.6 | 4.2 | 7.5 | 5.6 | |
| Ezetimibe | 3.8 | 5.6 | 2.2 | 2.0 | 2.4 | 7.2 | 2.2 | 3.0 | NA | 10.5 | 3.6 | |
| Anti-gout preparations | 13.5 | 12.5 | 13.6 | 14.9 | 14.1 | 10.6 | 12.5 | 13.9 | 13.6 | 12.3 | 17.1 | |
| Antithyroid Agent | 0.6 | NA | NA | NA | NA | NA | NA | NA | NA | NA | NA | |
| NSAIDs | 22.4 | 28.6 | 21.3 | 25.5 | 27.2 | 16.1 | 23.9 | 16.5 | 25.0 | 24.3 | 19.1 | |
| Antifungal drugs | 1.4 | NA | NA | NA | 1.9 | NA | 1.7 | 2.7 | NA | 2.4 | NA | |
| Antituberculosis agents | 0.2 | NA | 0.0^†^ | 0.0^†^ | NA | NA | NA | NA | 0.0^†^ | NA | NA | |
| Therapeutic agents for  chronic hepatitis B or C | 0.2 | 0.0^†^ | 0.0^†^ | NA | NA | NA | NA | NA | NA | NA | NA | |
| **First visit**^‡^ **(%)** | 3.4 | 6.4 | 4.2 | NA | 2.4 | 2.4 | 3.9 | 2.4 | 7.2 | 6.4 | 2.9 | |
| **Emergency care**^‡^ **(%)** | 14.4 | 5.6 | 6.4 | 19.5 | 21.8 | 12.4 | 17.4 | 18.3 | 9.2 | 7.9 | 10.8 | |

Some categories in which the patient numbers did not exceed 10 were not shown due to privacy.

Abbreviations: NSAID, non-steroidal anti-inflammatory drug.

NA is the case when the number of patients was less than 10.

† Not included in logistic regression model for assessing the association with missingness.

‡ At the date of the first prescription of any antidiabetic drug.

§ 180 days prior to the date of the first prescription of any antidiabetic drug.

# **Supplementary Table S4. Patient characteristics in the scenario 3 cohort:**

|  | All hospitals combined | Individual hospital | | | | | | | | | |
| --- | --- | --- | --- | --- | --- | --- | --- | --- | --- | --- | --- |
|  |  | 1 | 2 | 3 | 4 | 5 | 6 | 7 | 8 | 9 | 10 |
| **New users(n)** | 3,481 | 273 | 298 | 435 | 511 | 328 | 439 | 426 | 271 | 235 | 265 |
| **Sex, male**^‡^ **(%)** | 74.5 | 76.6 | 71.1 | 75.2 | 74.4 | 70.7 | 75.6 | 77.9 | 67.9 | 77.4 | 77.4 |
| **Age**^‡^, **mean (SD)** | 70.5 (13.1) | 70.5 (12.6) | 70.0 (12.8) | 72.6 (11.0) | 71.8 (13.1) | 68.7 (14.2) | 70.5 (12.3) | 72.2 (12.6) | 68.5 (15.2) | 70.3 (13.1) | 67.4 (14.5) |
| **Year of cohort entry**^‡^**(%)** |  |  |  |  |  |  |  |  |  |  |  |
| 2015 | 19.8 | 18.3 | 14.4 | 23.9 | 24.1 | 19.8 | 20.3 | 18.5 | 21.0 | 15.7 | 16.2 |
| 2016 | 39.6 | 37.7 | 35.6 | 39.8 | 35.8 | 42.4 | 42.1 | 39.9 | 40.2 | 41.7 | 42.6 |
| 2017 | 40.6 | 44.0 | 50.0 | 36.3 | 40.1 | 37.8 | 37.6 | 41.5 | 38.7 | 42.6 | 41.1 |
| **Hospitalization**^‡^ **(%)** | 32.1 | 16.1 | 19.5 | 38.9 | 41.1 | 37.2 | 36.0 | 39.7 | 15.9 | 29.4 | 28.7 |
| **Number of ambulatory medical visits^†§^**, **mean (SD)** | 7.5 (8.6) | 6.6 (4.2) | 7.5 (9.2) | 7.0 (8.7) | 7.7 (7.9) | 6.6 (4.7) | 8.4 (10.6) | 7.3 (7.3) | 6.4 (6.2) | 8.0 (10.0) | 8.9 (13.8) |
| **Number of hospitalizations^†§^**, **mean (SD)** | 0.6 (0.7) | 0.3 (0.6) | 0.4 (0.7) | 0.6 (0.8) | 0.6 (0.7) | 0.6 (0.6) | 0.7 (0.8) | 0.6 (0.8) | 0.4 (0.7) | 0.6 (0.8) | 0.5 (0.8) |
| **Class number of any prescriptions^†§^**, **mean (SD)** | 12 (7.8) | 11.5 (8.3) | 11.2 (7.4) | 12.9 (8.9) | 13.0 (7.3) | 12.1 (7.0) | 13.1 (8.0) | 11.0 (7.1) | 11.1 (7.9) | 12.5 (8.7) | 10.4 (6.8) |
| **Class number of concomitant medications^§^**, **mean (SD)** | 2.2 (1.4) | 2.4 (1.5) | 2.1 (1.5) | 2.1 (1.4) | 2.4 (1.5) | 2.3 (1.3) | 2.2 (1.4) | 2.0 (1.4) | 2.2 (1.4) | 2.4 (1.5) | 2.1 (1.4) |
| **Exposure (%)** |  |  |  |  |  |  |  |  |  |  |  |
| Allopurinol | 31.3 | 19.0 | 53.7 | 22.8 | 34.2 | 30.5 | 31.7 | 29.8 | 28.4 | 35.3 | 28.7 |
| Febuxostat | 68.7 | 81.0 | 46.3 | 77.2 | 65.8 | 69.5 | 68.3 | 70.2 | 71.6 | 64.7 | 71.3 |
| **Complications^§^ (%)** |  |  |  |  |  |  |  |  |  |  |  |
| Leukemia | 0.4 | 0.0^†^ | 0.0^†^ | NA | NA | NA | NA | NA | 0.0^†^ | 0.0^†^ | 0.0^†^ |
| Heart failure | 28.0 | 42.5 | 28.5 | 34.9 | 24.5 | 27.1 | 25.1 | 15.5 | 29.2 | 34.9 | 27.2 |
| Acute myocardial infarction | 10.5 | 19.8 | 4.7 | 11.5 | 6.3 | 12.5 | 9.3 | 8.9 | 12.2 | 14.9 | 10.9 |
| Hypertension | 16.6 | 19.8 | 12.1 | 13.8 | 19.2 | 10.7 | 18.0 | 20.9 | 17.0 | 20.0 | 12.5 |
| Cerebrovascular diseases | 6.7 | 7.7 | 5.0 | 10.6 | 9.8 | 5.8 | 8.9 | NA | NA | 8.5 | 3.8 |
| Hyperlipidemia | 13.3 | 12.8 | 13.8 | 12.2 | 12.1 | 12.2 | 9.6 | 17.6 | 10.3 | 20.0 | 15.1 |
| Diabetes mellitus | 41.9 | 50.5 | 30.5 | 40.0 | 40.1 | 43.6 | 40.3 | 51.9 | 48.0 | 36.2 | 35.5 |
| Renal failure | 12.2 | 8.8 | 12.1 | 14.5 | 11.5 | 22.3 | 12.5 | 10.6 | 7.4 | 9.8 | 10.6 |
| Other liver disease | 5.3 | NA | NA | 9.0 | 7.0 | 5.8 | 4.8 | 6.6 | 4.8 | NA | NA |
| Malignant tumors | 25.8 | 16.1 | 21.5 | 28.0 | 35.0 | 19.5 | 28.2 | 37.6 | 15.5 | 14.9 | 23.8 |
| **Concomitant medication^§^ (%)** |  |  |  |  |  |  |  |  |  |  |  |
| Other anti-gout preparations | 2.6 | NA | NA | NA | 2.2 | NA | 2.5 | 2.3 | NA | 4.3 | 5.7 |
| NSAIDs | 24.7 | 23.8 | 26.5 | 24.6 | 32.5 | 20.1 | 21.0 | 18.3 | 30.6 | 30.6 | 19.2 |
| ARB | 47.1 | 56.0 | 46.3 | 43.2 | 44.6 | 55.5 | 43.7 | 46.9 | 44.3 | 49.8 | 45.3 |
| ACE inhibitors | 10.3 | 9.2 | 5.7 | 6.0 | 7.0 | 12.8 | 11.2 | 12.9 | 9.6 | 14.5 | 18.9 |
| Beta-blockers | 32.9 | 35.2 | 25.8 | 40.7 | 30.7 | 42.1 | 36.7 | 27.2 | 21.4 | 39.1 | 27.9 |
| Calcium channel blockers | 54.2 | 58.2 | 55.7 | 54.7 | 53.6 | 56.4 | 54.9 | 46.9 | 61.6 | 49.8 | 53.2 |
| Diuretics | 43.8 | 43.6 | 37.2 | 46.9 | 50.3 | 47.9 | 47.8 | 37.8 | 41.3 | 42.1 | 35.1 |
| New quinolone antibiotic | 7.4 | 5.5 | 10.7 | 7.1 | 10.4 | NA | 7.3 | 7.0 | 4.8 | 10.6 | 6.8 |
| Aminoglycoside antibiotic | 0.5 | NA | NA | NA | NA | 0.0^†^ | NA | NA | 0.0^†^ | 0.0^†^ | NA |
| **First visit**^‡^ **(%)** | 2.2 | NA | 3.4 | NA | 3.1 | NA | NA | NA | 5.2 | NA | NA |
| **Emergency care**^‡^ **(%)** | 12.0 | 5.5 | 9.1 | 17.0 | 22.9 | 5.8 | 13.0 | 11.5 | 5.5 | 9.4 | 9.1 |

Some categories in which the patient numbers did not exceed 10 were not shown due to privacy.

Abbreviations: NSAID, non-steroidal anti-inflammatory drug; ARB, angiotensin receptor blocker; ACE, angiotensin-converting enzyme.

NA is the case when the number of patients was less than 10.

† Not included in logistic regression model for assessing the association with missingness.

‡ At the date of the first prescription of any antidiabetic drug.

§ 180 days prior to the date of the first prescription of any antidiabetic drug.

# **Supplementary Table S5. Patient characteristics in the scenario 4 cohort:**

|  | All hospitals combined | Individual hospital | | | | | | | | | |
| --- | --- | --- | --- | --- | --- | --- | --- | --- | --- | --- | --- |
|  |  | 1 | 2 | 3 | 4 | 5 | 6 | 7 | 8 | 9 | 10 |
| **New users(n)** | 10,372 | 738 | 781 | 1,471 | 1,385 | 1,109 | 1,572 | 1,294 | 602 | 680 | 740 |
| **Sex, male**^‡^ **(%)** | 52.9 | 49.9 | 49.6 | 54.7 | 53.6 | 52.9 | 54.3 | 54.1 | 46.8 | 56.6 | 50.4 |
| **Age**^‡^, **mean (SD)** | 67.4 (16) | 68.4 (15.8) | 68.9 (15.2) | 67.5 (15.4) | 68.5 (15.2) | 65.0 (17.1) | 66.4 (15.9) | 69.3 (15.1) | 64.2 (18.2) | 68.9 (16.6) | 65.3 (16.6) |
| **Year of cohort entry**^‡^**(%)** |  |  |  |  |  |  |  |  |  |  |  |
| 2016 | 34.4 | 33.1 | 30.6 | 35.8 | 35.3 | 40.0 | 32.8 | 33.1 | 37.0 | 31.2 | 34.2 |
| 2017 | 65.6 | 66.9 | 69.4 | 64.2 | 64.7 | 60.0 | 67.2 | 66.9 | 63.0 | 68.8 | 65.8 |
| **Hospitalization**^‡^ **(%)** | 36.6 | 27.1 | 32.5 | 38.8 | 41.2 | 39.3 | 35.7 | 40.6 | 27.2 | 38.8 | 33.2 |
| **Number of ambulatory medical visits^†§^**, **mean (SD)** | 7.4 (8.6) | 6.9 (5) | 7.2 (8.5) | 7 (8.5) | 8 (8) | 6.8 (5.9) | 7.7 (9.8) | 7.4 (8.3) | 7.2 (10.5) | 8.3 (10.7) | 7.9 (10.4) |
| **Number of hospitalizations^†§^**, **mean (SD)** | 0.6 (0.8) | 0.4 (0.7) | 0.5 (0.7) | 0.6 (0.7) | 0.6 (0.8) | 0.6 (0.8) | 0.6 (0.7) | 0.7 (0.8) | 0.4 (0.7) | 0.6 (0.8) | 0.5 (0.8) |
| **Class number of any prescriptions^†§^**, **mean (SD)** | 11.2 (7.7) | 11 (7.9) | 10.6 (6.8) | 11.8 (9.7) | 12.1 (8) | 10.9 (6.7) | 11.5 (7.7) | 10.6 (7.1) | 10.7 (7) | 11.4 (7.1) | 9.8 (6.7) |
| **Class number of concomitant medications^§^**, **mean (SD)** | 1.6 (1.5) | 1.6 (1.6) | 1.6 (1.5) | 1.6 (1.6) | 1.8 (1.6) | 1.6 (1.4) | 1.7 (1.6) | 1.5 (1.5) | 1.7 (1.5) | 1.7 (1.5) | 1.5 (1.4) |
| **Exposure (%)** |  |  |  |  |  |  |  |  |  |  |  |
| Other PPIs | 60.7 | 70.5 | 44.4 | 73.4 | 48.5 | 66.5 | 73.5 | 63.8 | 77.7 | 34.9 | 34.3 |
| Lansoprazole | 39.3 | 29.5 | 55.6 | 26.6 | 51.5 | 33.5 | 26.5 | 36.2 | 22.3 | 65.1 | 65.7 |
| **Complications^§^ (%)** |  |  |  |  |  |  |  |  |  |  |  |
| Renal failure | 4.0 | 5.3 | 3.8 | 6.1 | 3.9 | 4.1 | 3.5 | 2.9 | 3.8 | 4.1 | 2.7 |
| Liver cirrhosis | 1.9 | NA | 1.7 | 1.3 | 3.2 | 2.8 | 1.3 | 3.1 | NA | NA | NA |
| Pancreatitis | 3.2 | 1.4 | 2.2 | 2.7 | 1.3 | 3.9 | 3.6 | 1.4 | 19.3 | 1.6 | NA |
| Heart failure | 19.1 | 27.8 | 19.2 | 18.6 | 19.7 | 16.2 | 19.9 | 13.0 | 18.8 | 27.1 | 16.9 |
| Inflammatory bowel disease | 0.4 | 0.0^†^ | NA | 0.8 | NA | NA | NA | NA | 2.2 | NA | NA |
| Adrenal insufficiency | 0.3 | 0.0^†^ | 0.0^†^ | NA | 0.7 | NA | NA | NA | 0.0^†^ | NA | 0.0^†^ |
| Hypothyroidism | 5.7 | 10.2 | 2.9 | 3.9 | 2.5 | 9.6 | 8.8 | 1.7 | 11.3 | 2.1 | 6.5 |
| Helicobacter pylori infection | 11.5 | 15.0 | 11.8 | 9.7 | 12.2 | 12.9 | 13.4 | 9.4 | 8.6 | 10.1 | 10.7 |
| Other liver disease | 3.4 | 3.7 | 3.3 | 3.1 | 4.8 | 3.2 | 3.6 | 3.5 | 3.2 | 2.8 | 1.8 |
| **Concomitant medication^§^ (%)** |  |  |  |  |  |  |  |  |  |  |  |
| Antiepileptic drugs | 0.8 | NA | NA | 0.7 | 0.9 | 1.5 | 0.7 | 0.9 | NA | NA | NA |
| ARB | 24.8 | 28.9 | 28.6 | 21.9 | 24.8 | 27.9 | 21.4 | 25.2 | 22.8 | 24.0 | 27.3 |
| ACE inhibitors | 4.6 | 3.4 | 2.7 | 2.4 | 2.5 | 6.9 | 5.8 | 4.6 | 6.0 | 7.9 | 5.7 |
| Diuretics | 17.7 | 15.0 | 16.4 | 19.6 | 20.6 | 18.0 | 19.1 | 16.8 | 15.6 | 16.6 | 12.8 |
| Antidepressants | 3.2 | 4.2 | 2.3 | 3.4 | 2.3 | 5.5 | 2.7 | 3.2 | 3.7 | NA | 2.7 |
| Antipsychotics | 4.0 | 4.5 | 2.3 | 3.8 | 4.4 | 4.5 | 4.6 | 4.9 | 3.2 | 4.0 | 2.3 |
| Corticosteroids | 12.8 | 5.4 | 8.3 | 10.4 | 13.6 | 12.5 | 16.9 | 16.5 | 10.5 | 14.1 | 13.6 |
| NSAIDs | 28.8 | 32.5 | 33.3 | 30.3 | 32.8 | 25.6 | 24.6 | 22.3 | 34.2 | 30.3 | 29.1 |
| Anti-gout preparations | 9.5 | 9.8 | 11.7 | 8.4 | 10.5 | 6.9 | 10.2 | 9.3 | 10.5 | 8.2 | 10.1 |
| Beta blocker | 16.3 | 13.3 | 11.5 | 20.0 | 15.3 | 16.9 | 19.5 | 12.3 | 12.1 | 24.3 | 13.6 |
| Calcium antagonist | 35.1 | 38.8 | 39.6 | 35.3 | 36.2 | 34.2 | 35.8 | 30.3 | 39.2 | 31.5 | 32.2 |
| Aminoglycoside antibiotic | 6.8 | 8.5 | 8.2 | 6.9 | 9.2 | 2.4 | 6.8 | 6.3 | 5.5 | 8.5 | 6.2 |
| New quinolone antibiotic | 0.5 | NA | NA | NA | NA | 0.0^†^ | NA | 1.0 | NA | NA | NA |

Some categories in which the patient numbers did not exceed 10 were not shown due to privacy.

Abbreviations: PPI, proton pump inhibitor; ARB, angiotensin receptor blocker; ACE, angiotensin-converting enzyme; NSAID, non-steroidal anti-inflammatory drug.

NA is the case when the number of patients was less than 10.

† Not included in logistic regression model for assessing the association with missingness.

‡ At the date of the first prescription of any antidiabetic drug.

§ 180 days prior to the date of the first prescription of any antidiabetic drug.

# **Supplementary Table S6. Patient characteristics in the scenario 5 cohort:**

|  | All hospitals combined | Individual hospital | | | | | | | | | |
| --- | --- | --- | --- | --- | --- | --- | --- | --- | --- | --- | --- |
|  |  | 1 | 2 | 3 | 4 | 5 | 6 | 7 | 8 | 9 | 10 |
| **New users(n)** | 2,994 | 218 | 197 | 350 | 513 | 405 | 367 | 390 | 166 | 188 | 200 |
| **Sex, male**^‡^ **(%)** | 61.5 | 55.5 | 57.4 | 71.1 | 62.0 | 64.4 | 59.9 | 61.8 | 50.0 | 59.6 | 62.0 |
| **Age**^‡^, **mean (SD)** | 69.4 (12.4) | 70.6 (11.1) | 69.8 (10.9) | 71.5 (11.1) | 69.6 (12.7) | 68.4 (12.5) | 68.7 (13) | 68.8 (13.2) | 69.5 (12.7) | 71.7 (10.6) | 66.1 (13.6) |
| **Year of cohort entry**^‡^**(%)** |  |  |  |  |  |  |  |  |  |  |  |
| 2015 | 21.2 | 22.0 | 21.8 | 15.7 | 24.6 | 23.5 | 22.6 | 20.5 | 24.1 | 19.1 | 14.0 |
| 2016 | 42.6 | 40.4 | 44.7 | 45.1 | 44.2 | 41.2 | 42.5 | 43.6 | 42.8 | 38.8 | 38.0 |
| 2017 | 36.3 | 37.6 | 33.5 | 39.1 | 31.2 | 35.3 | 34.9 | 35.9 | 33.1 | 42.0 | 48.0 |
| **Hospitalization**^‡^ **(%)** | 35.7 | 19.7 | 23.4 | 51.4 | 35.1 | 36.0 | 35.4 | 43.1 | 24.1 | 35.1 | 35.0 |
| **Number of ambulatory medical visits^†§^**, **mean (SD)** | 7.1 (8.2) | 6.8 (4.8) | 7.2 (9.6) | 7.2 (9.7) | 7.2 (6.1) | 6.7 (6.6) | 7.5 (9.9) | 6.4 (6) | 7.1 (10.1) | 6.6 (6.8) | 8.5 (13.4) |
| **Number of hospitalizations^†§^**, **mean (SD)** | 0.5 (0.7) | 0.3 (0.6) | 0.4 (0.7) | 0.8 (0.8) | 0.5 (0.7) | 0.6 (0.7) | 0.6 (0.7) | 0.6 (0.7) | 0.4 (0.6) | 0.6 (0.7) | 0.5 (0.8) |
| **Class number of any prescriptions^†§^**, **mean (SD)** | 11.3 (7.8) | 11.5 (8.6) | 9.9 (6.6) | 13.3 (8.6) | 12 (8) | 11.7 (7.9) | 11.3 (8) | 10.1 (6.8) | 10.4 (7.6) | 11.4 (7.9) | 9.6 (7.4) |
| **Class number of concomitant medications^§^**, **mean (SD)** | 2 (1.6) | 2.3 (1.7) | 1.7 (1.4) | 2.1 (1.4) | 2.3 (1.7) | 2.2 (1.6) | 1.9 (1.5) | 1.8 (1.5) | 1.6 (1.4) | 1.9 (1.4) | 1.7 (1.5) |
| **Exposure (%)** |  |  |  |  |  |  |  |  |  |  |  |
| Other oral antidiabetic agents | 31.8 | 39.0 | 25.4 | 27.4 | 36.8 | 27 | 33.0 | 36.4 | 17.5 | 31.9 | 35.5 |
| DPP-4I | 68.2 | 61.0 | 74.6 | 72.6 | 63.2 | 73.1 | 67.0 | 63.6 | 82.5 | 68.1 | 64.5 |
| **Complications^§^ (%)** |  |  |  |  |  |  |  |  |  |  |  |
| Alcoholic pancreatitis | NA | 0.0^†^ | 0.0^†^ | 0.0^†^ | 0.0^†^ | 0.0^†^ | 0.0^†^ | NA | 0.0^†^ | 0.0^†^ | 0.0^†^ |
| Inflammatory bowel disease | NA | 0.0^†^ | 0.0^†^ | 0.0^†^ | 0.0^†^ | NA | NA | 0.0^†^ | 0.0^†^ | NA | 0.0 |
| Peptic ulcer disease | 6.0 | 5.0 | NA | 8.9 | 12.3 | 2.5 | 6.8 | 5.9 | NA | 5.3 | NA |
| Gallstone disease | 2.2 | NA | NA | 3.1 | 2.3 | NA | NA | 3.1 | NA | NA | NA |
| Acute appendicitis | NA | 0.0^†^ | 0.0^†^ | NA | 0.0^†^ | NA | 0.0^†^ | NA | 0.0^†^ | 0.0^†^ | 0.0^†^ |
| Diverticulitis | 1.1 | NA | NA | NA | NA | NA | NA | NA | NA | NA | 0.0^†^ |
| Pancreatic cancer | 3.0 | NA | NA | 4.3 | 5.5 | NA | 5.4 | NA | 0.0^†^ | 0.0^†^ | NA |
| Cholangiocarcinoma | 0.6 | NA | 0.0^†^ | NA | NA | NA | 0.0^†^ | NA | 0.0^†^ | 0.0^†^ | NA |
| Duodenal cancer | NA | 0.0^†^ | 0.0^†^ | NA | NA | 0.0^†^ | NA | NA | 0.0^†^ | 0.0^†^ | 0.0^†^ |
| Chronic kidney disease | 3.7 | NA | NA | 5.7 | 4.1 | 4.0 | 3.3 | NA | NA | NA | NA |
| Hepatitis | 2.6 | NA | NA | 2.9 | 2.9 | 4.0 | NA | NA | NA | 5.3 | NA |
| Liver cirrhosis | 2.0 | NA | NA | NA | NA | 2.7 | NA | 3.8 | NA | NA | 0.0^†^ |
| Fatty liver | 2.3 | NA | NA | NA | 2.3 | NA | 2.7 | 4.6 | NA | NA | NA |
| Alcoholic liver disease | NA | 0.0^†^ | 0.0^†^ | NA | 0.0^†^ | 0.0^†^ | NA | 0.0^†^ | 0.0^†^ | 0.0^†^ | 0.0^†^ |
| Heart failure | 22.1 | 32.6 | 23.4 | 29.4 | 23.8 | 18.5 | 18.5 | 12.6 | 19.3 | 35.1 | 15.5 |
| Acute myocardial infarction | 8.8 | 13.8 | 7.1 | 12.9 | 8.0 | 7.7 | 4.9 | 7.2 | 9.0 | 16.0 | 5.0 |
| Hypertension | 16.4 | 17.9 | 14.2 | 13.4 | 20.1 | 13.1 | 19.9 | 18.7 | 8.4 | 19.7 | 12.0 |
| Cerebrovascular diseases | 8.2 | 7.3 | 6.6 | 14.6 | 8.6 | 9.1 | 8.4 | 5.9 | NA | 7.4 | 5.0 |
| Hyperlipidemia | 16.1 | 16.5 | 17.8 | 14.9 | 15.0 | 13.3 | 18.3 | 17.4 | 10.8 | 25.5 | 13.5 |
| Peripheral vascular disease | 0.9 | NA | 0.0^†^ | NA | NA | NA | NA | 0.0^†^ | NA | NA | NA |
| Diabetic complication | 7.1 | 10.1 | 7.6 | NA | 6.4 | 9.4 | 10.1 | 9.0 | NA | 9.6 | NA |
| **Concomitant medication^§^ (%)** |  |  |  |  |  |  |  |  |  |  |  |
| Antiepileptic drugs | 0.6 | NA | 0.0^†^ | NA | NA | NA | NA | NA | NA | NA | NA |
| ARB | 39.6 | 47.2 | 38.6 | 36.9 | 38.0 | 44.2 | 40.1 | 37.7 | 38.0 | 35.6 | 40.5 |
| ACE inhibitors | 8.7 | 6.4 | NA | 8.3 | 6.0 | 13.1 | 10.1 | 6.9 | 6.0 | 17.0 | 9.5 |
| Diuretics | 24.1 | 23.4 | 16.2 | 29.1 | 31.0 | 28.1 | 23.2 | 18.2 | 18.7 | 20.7 | 19.0 |
| Antiarrhythmics Class I and III | 3.1 | NA | NA | 5.1 | 3.1 | 4.2 | 3.0 | NA | NA | NA | NA |
| Thiazolidinediones | 4.7 | 14.7 | 5.6 | 3.4 | 4.9 | 3.2 | 3.5 | 3.1 | NA | 7.4 | NA |
| Glinides | 4.1 | NA | NA | 2.9 | 7.8 | 3.7 | 3.3 | 2.8 | NA | NA | NA |
| SGLT2 inhibitors | 2.9 | NA | NA | NA | 3.5 | NA | 2.7 | 4.4 | NA | NA | 5.0 |
| Insulin | 22.6 | 18.3 | 21.3 | 26.3 | 32.4 | 27.4 | 18.0 | 27.7 | NA | 10.1 | 14.5 |
| GLP-1 receptor agonists | 1.3 | 5.0 | NA | NA | NA | NA | NA | NA | 0.0^†^ | 0.0^†^ | NA |
| Corticosteroids | 9.1 | 5.5 | 7.1 | 6.3 | 10.5 | 10.9 | 9.8 | 12.3 | 6.6 | 9.0 | 7.0 |
| Estrogen | NA | 0.0^†^ | NA | 0.0^†^ | NA | 0.0^†^ | 0.0^†^ | 0.0^†^ | 0.0^†^ | NA | 0.0 |
| NSAIDs | 20.7 | 24.8 | 17.3 | 22.6 | 25.1 | 16.5 | 18.3 | 17.7 | 25.3 | 22.9 | 18.0 |
| Codeine | 1.1 | NA | NA | NA | NA | NA | NA | NA | NA | NA | NA |
| Proton pump inhibitors | 39.2 | 40.8 | 38.1 | 52.6 | 38.6 | 45.7 | 36.5 | 32.3 | 25.9 | 36.7 | 36.0 |
| H2 antagonists | 11.0 | 15.1 | 11.2 | 8.6 | 13.1 | 8.1 | 11.2 | 9.5 | 15.1 | 13.8 | 8.0 |
| 5-aminosalicylic acid agents | 0.5 | NA | NA | NA | NA | NA | NA | NA | 0.0^†^ | NA | NA |
| **Examination / operation** |  |  |  |  |  |  |  |  |  |  |  |
| ERCP | NA | 0.0^†^ | 0.0^†^ | NA | 0.0^†^ | 0.0^†^ | NA | 0.0^†^ | 0.0^†^ | 0.0^†^ | 0.0^†^ |
| Biliary tract operation | NA | 0.0^†^ | 0.0^†^ | 0.0^†^ | NA | NA | NA | 0.0^†^ | 0.0^†^ | 0.0^†^ | 0.0^†^ |
| Gastrectomy | NA | 0.0^†^ | 0.0^†^ | 0.0^†^ | 0.0^†^ | NA | 0.0^†^ | 0.0^†^ | 0.0^†^ | 0.0^†^ | 0.0^†^ |
| **First visit, emergency care (%)** | 2.3 | NA | NA | NA | 2.1 | 4.2 | NA | NA | NA | NA | NA |
| **Emergency care (%)** | 11.3 | 6.9 | 9.1 | 24.0 | 11.9 | 6.4 | 12.3 | 12.6 | 7.8 | 8.0 | 6.5 |

Some categories in which the patient numbers did not exceed 10 were not shown due to privacy.

Abbreviations: DPP-4I; dipeptidyl peptidase-4 inhibitors; ARB, angiotensin receptor blocker; ACE, angiotensin-converting enzyme; SGLT2, sodium glucose cotransporter 2; GLP-1, glucagon like peptide-1; NSAID, non-steroidal anti-inflammatory drug; ERCP, endoscopic retrograde cholangiopancreatography.

NA is the case when the number of patients was less than 10.

† Not included in logistic regression model for assessing the association with missingness.

‡ At the date of the first prescription of any antidiabetic drug.

§ 180 days prior to the date of the first prescription of any antidiabetic drug.

**
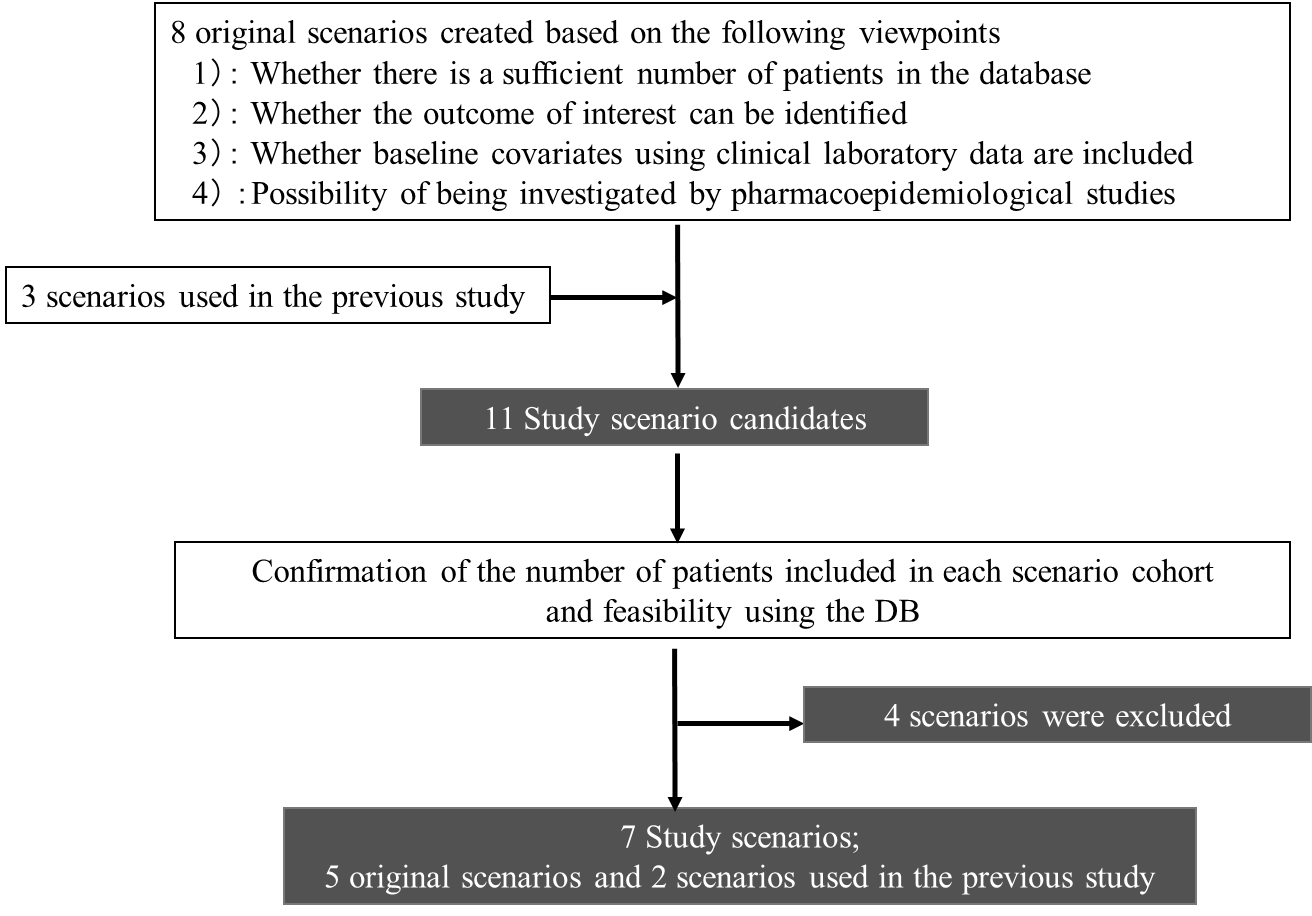
**

# **Supplementary Figure S6. Study scenario selection flowchart.**

7 cohort study scenarios were created from 11 study scenario candidates. Abbreviations: DB, database.

**
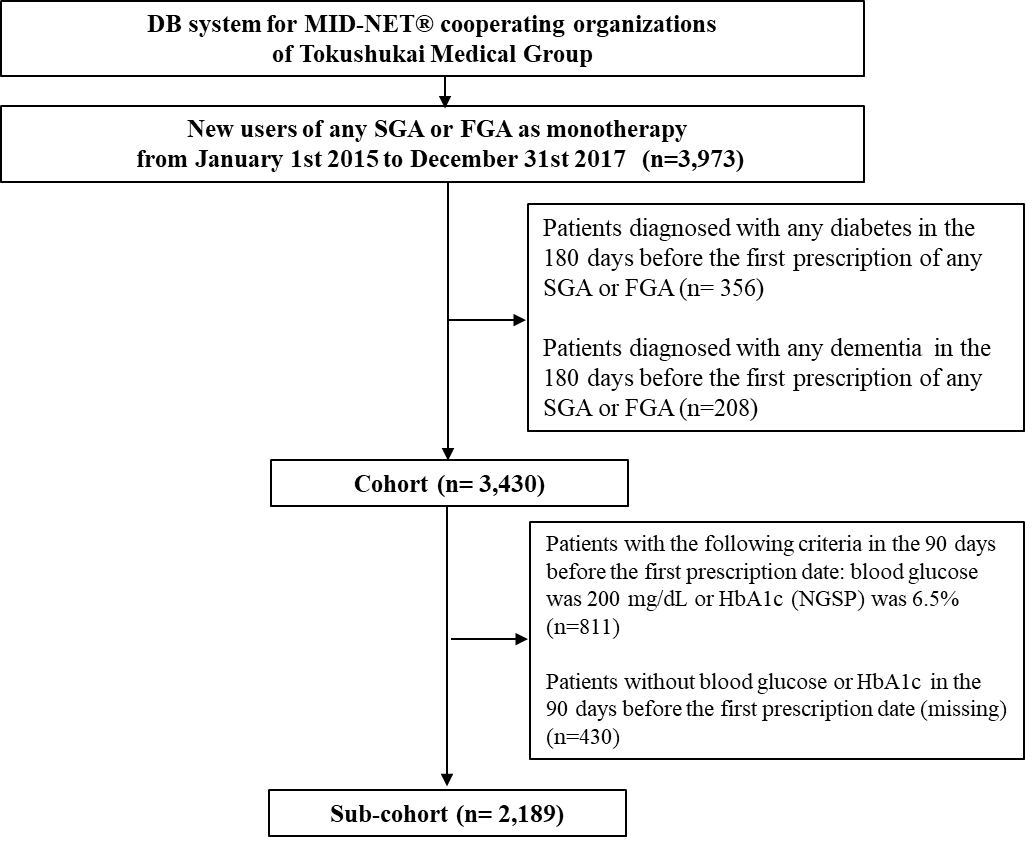
**

# **Supplementary Figure S7. Number of patients in the study cohort of scenario 1: risk of diabetes associated with antipsychotic drugs.**

3,430 new antipsychotics users were included in the study cohort of scenario 1. Abbreviations: DB, database; SGA, second-generation antipsychotic; FGA, first-generation antipsychotic.


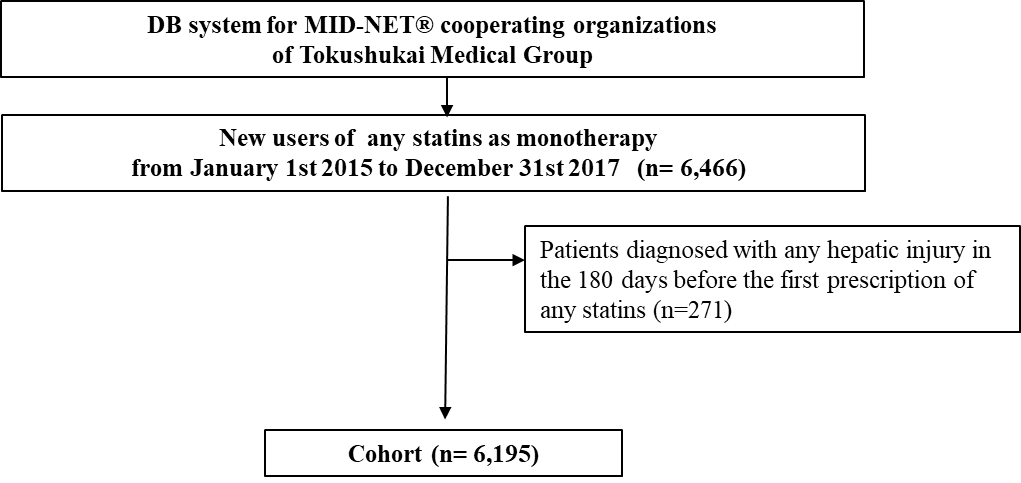


# **Supplementary Figure S8. Number of patients in the study cohort of scenario 2: risk of hepatic injury associated with statin use.**

6,195 new statin users were included in the study cohort of scenario 2. Abbreviations: DB, database.


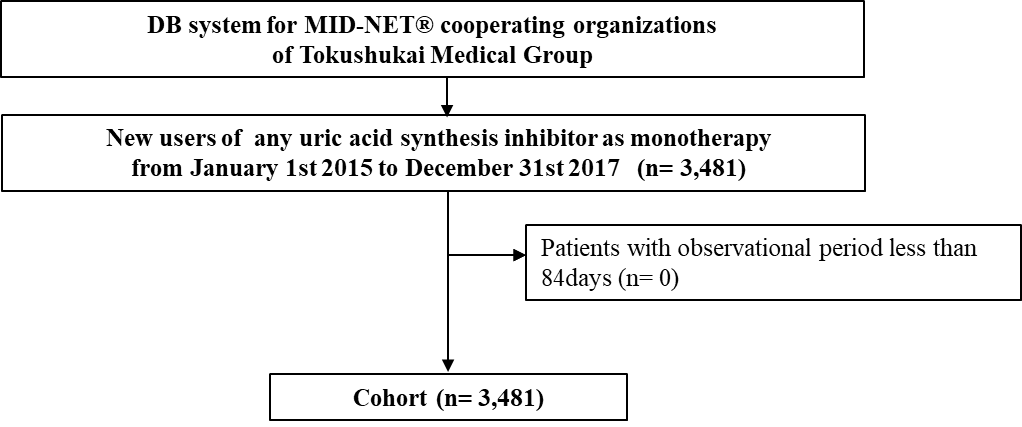


# **Supplementary Figure S9. Number of patients in the study cohort of scenario 3: effect of uric acid reduction by uric acid synthesis inhibitor use.**

3,481 new users of uric acid synthesis inhibitors were included in the study cohort of scenario 3. Abbreviations: DB, database.


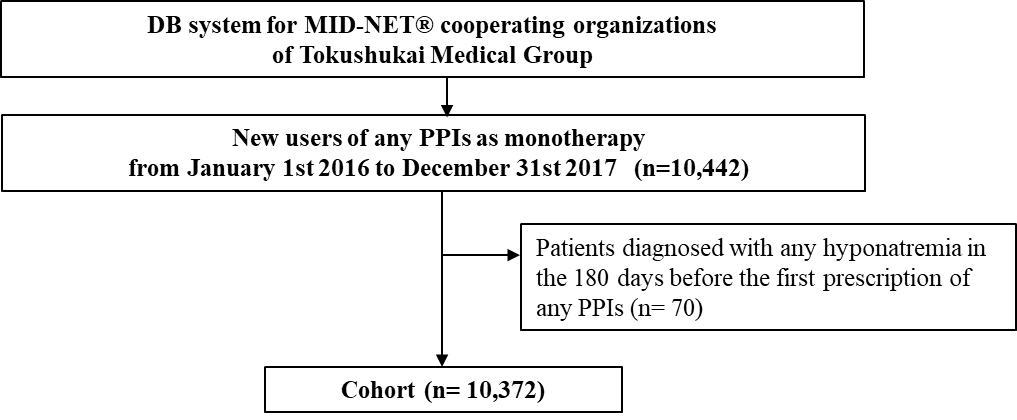


# **Supplementary Figure S10. Number of patients in the study cohort of scenario 4: risk of hyponatremia associated with proton-pump inhibitor use.**

10,372 new PPI users were included in the study cohort of scenario 4. Abbreviations: DB, database; PPI, proton pump inhibitor.


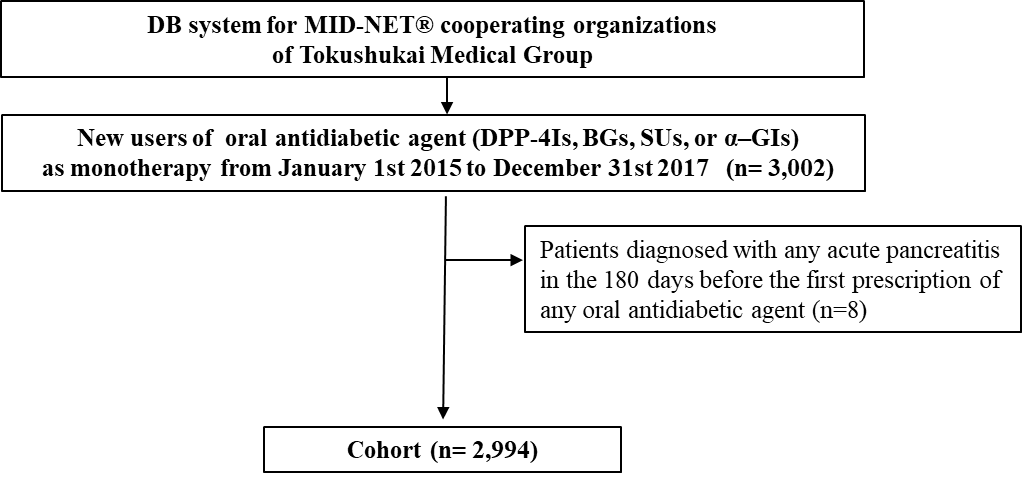


# **Supplementary Figure S11. Number of patients in the study cohort of scenario 5: risk of acute pancreatitis associated with oral antidiabetic drug use.**

2,994 new users of oral antidiabetics were included in the study cohort of scenario 5. Abbreviations: DB, database; DPP-4I, dipeptidyl peptidase-4 inhibitor; BG, biguanide; SU, sulfonylurea; α-GI, α-glucosidase inhibitor.


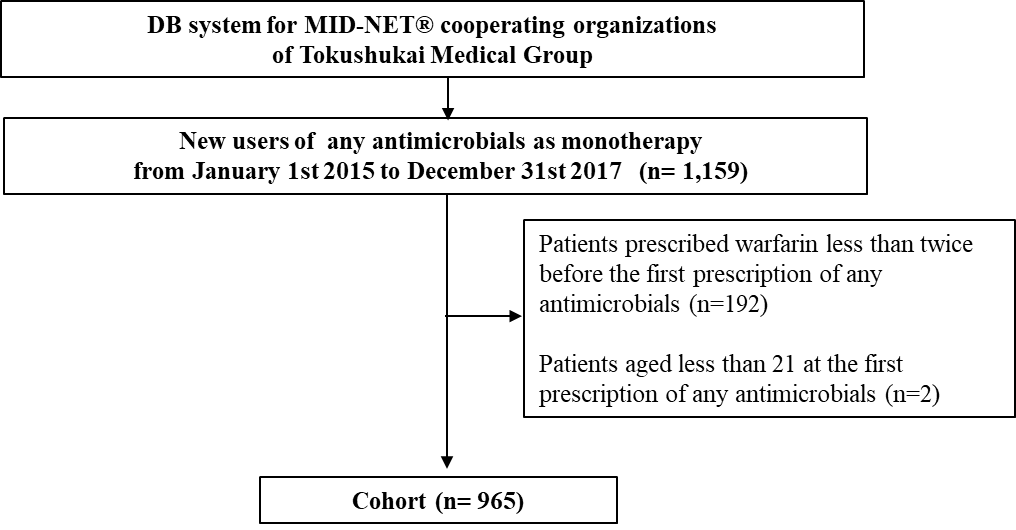


# **Supplementary Figure S12. Number of patients in the study cohort of scenario 6: risk of bleeding associated with combination use of warfarin and antimicrobial.**

965 new users of combinations of antimicrobials with warfarin were included in the study cohort of scenario 6. Abbreviations: DB, database.


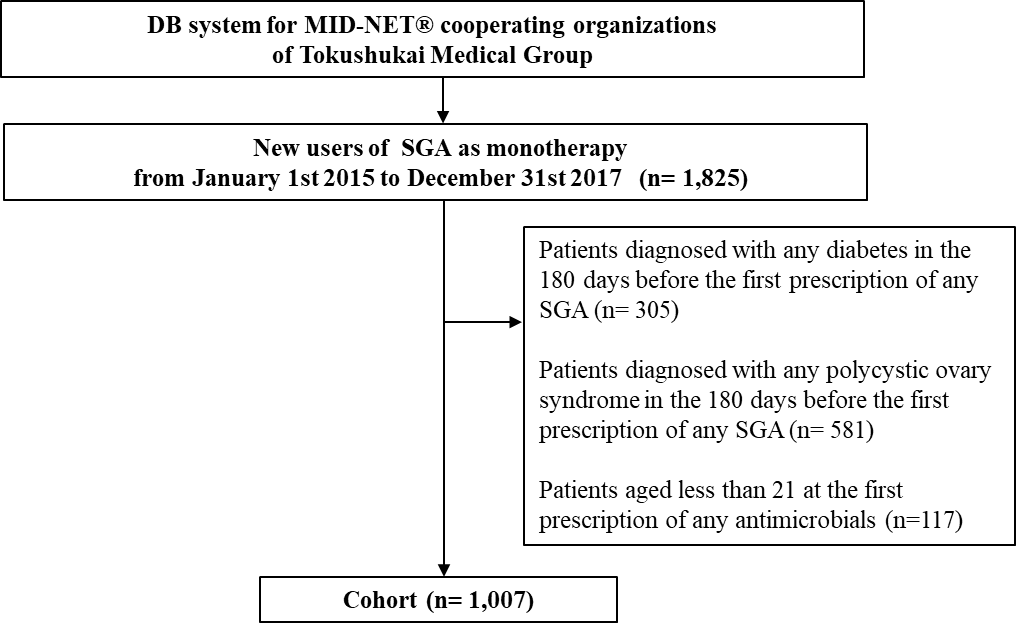


# **Supplementary Figure S13. Number of patients in the study cohort of scenario 7: risk of diabetes associated with SGA.**

1,007 new SGA users were included in the study cohort of scenario 7. Abbreviations: DB, database; SGA, second-generation antipsychotic.


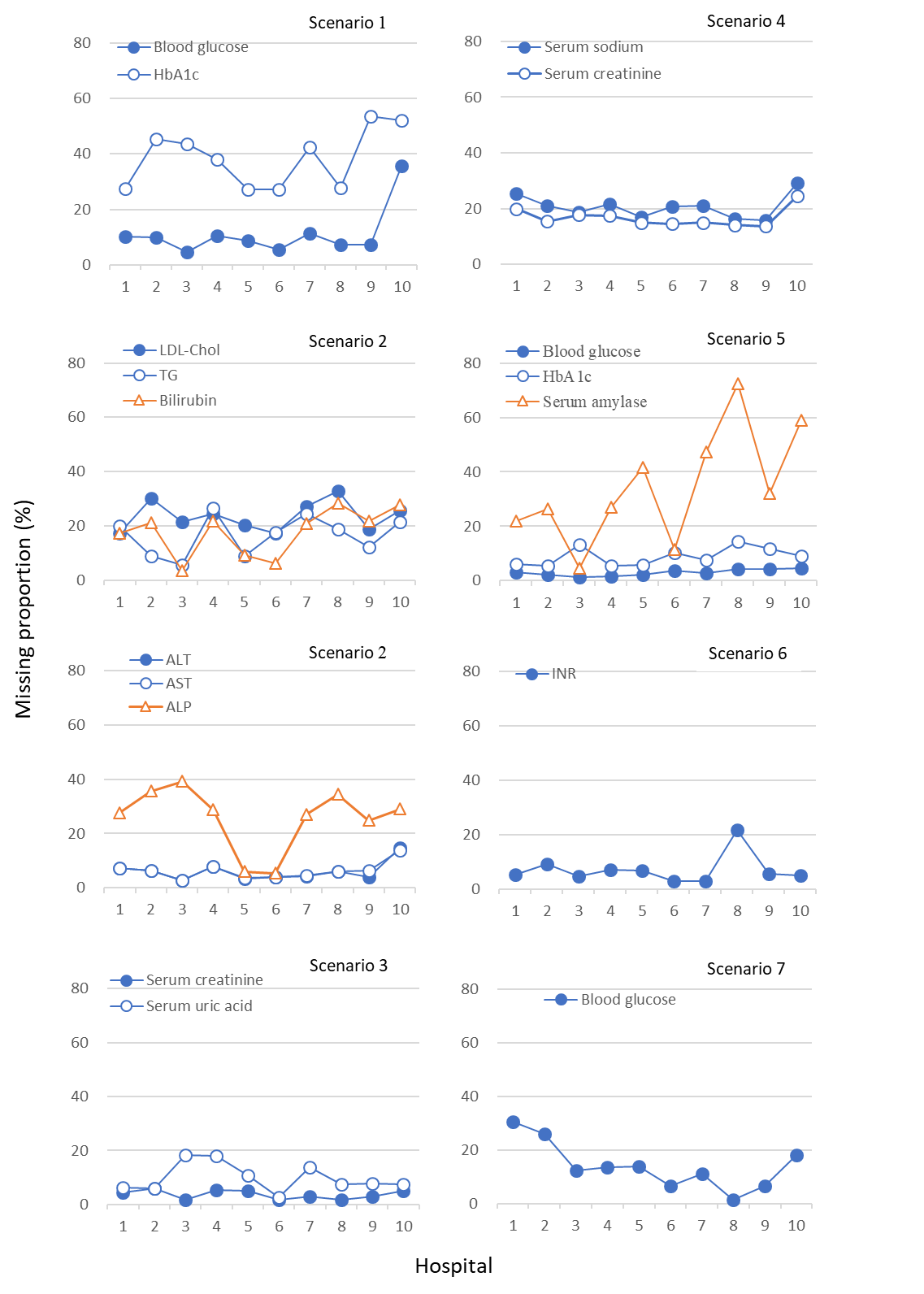


# **Supplementary Figure S14. Missing proportion in 180 days before the prescription in each hospital.**

This figure describes the differences in missing proportion by hospital when extending the target period to 180 days. Abbreviations: HbA1c, hemoglobin A1c; LDL-chol, low-density lipoprotein cholesterol; TG, triglyceride; ALT, alanine transaminase; AST, aspartate aminotransferase; ALP, alkaline phosphatase; INR, international normalized ratio.
